# Optimising error rates in programmes of pilot and definitive trials using Bayesian statistical decision theory

**DOI:** 10.1177/09622802251322987

**Published:** 2025-03-31

**Authors:** Duncan T Wilson, Andrew Hall, Julia M Brown, Rebecca EA Walwyn

**Affiliations:** Leeds Institute of Clinical Trials Research, 4468 University of Leeds, UK

**Keywords:** Clinical trial, pilot trial, external pilot, statistical decision theory, optimal design, expected utility

## Abstract

Pilot trials are often conducted in advance of definitive trials to assess their feasibility and to inform their design. Although pilot trials typically collect primary endpoint data, preliminary tests of effectiveness have been discouraged given their typically low power. Power could be increased at the cost of a higher type I error rate, but there is little methodological guidance on how to determine the optimal balance between these operating characteristics. We consider a Bayesian decision-theoretic approach to this problem, introducing a utility function and defining an optimal pilot and definitive trial programme as that which maximises expected utility. We base utility on changes in average primary outcome, the cost of sampling, treatment costs, and the decision-maker’s attitude to risk. We apply this approach to re-design OK-Diabetes, a pilot trial of a complex intervention with a continuous primary outcome with known standard deviation. We then examine how optimal programme characteristics vary with the parameters of the utility function. We find that the conventional approach of not testing for effectiveness in pilot trials can be considerably sub-optimal.

## 1. Introduction

Randomised pilot trials are a type of feasibility study which take the same form as a planned definitive randomised clinical trial, but on a smaller scale.^1^ Internal pilots constitute the initial phase of the definitive trial, with the pilot data being used in the final analysis. In contrast, external pilots are conducted separately to the definitive trial, with a clear gap between the two stages. A key goal of any pilot trial is to guide the decision of whether or not the definitive trial should go ahead, typically with a focus on feasibility issues such as recruitment rates and levels of missing data.^2–4^

Randomised pilot trials generally collect data measuring the effectiveness of the intervention, and this could be used to inform the decision of progression to the definitive trial. However, several authors have discouraged assessing effectiveness at the pilot stage due to concerns that the small pilot sample size will provide low power and lead to effective interventions being incorrectly discarded.^5–8^ This criticism rests on two assumptions. Firstly, it assumes that the pilot and definitive trials will share a primary endpoint. Secondly, it assumes that any pilot trial hypothesis test will be conducted with a significance level in the conventional range of 0.01–0.1. For example, consider a two-arm parallel group external pilot trial with a normally distributed primary endpoint and 35 participants per arm, as suggested by Teare et al.^9^ when the goal of the pilot trial is to estimate the standard deviation of the outcome. This would have a power of 23% (or equivalently, a type II error of β=0.77 ) to detect a standardised effect size of 0.3 when using a one-sided type I error rate of α=0.025 .

**Figure 1.**
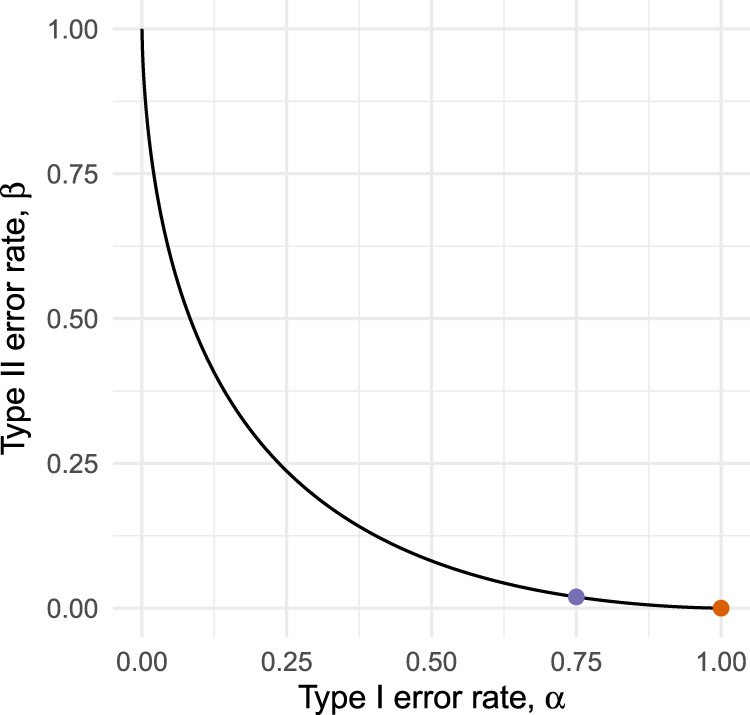
Operating characteristic curves for a hypothetical external pilot trial with fixed sample size testing efficacy.

While the assumption of a shared primary endpoint will often hold, there is no obvious reason for type I error rates in pilots to be constrained at conventionally low levels. Indeed, by not testing at all we effectively obtain a procedure with error rates α=1,β=0 . This testing strategy is only optimal if we have an absolute preference for minimising type II errors over type I errors in the pilot, a preference too extreme to be expected in practice. As illustrated in Figure 1, it will often be possible to reduce α considerably (in our example, from 1 to 0.75) at the cost of only a small increase in β (from 0 to 0.027). Although relaxing the type I error rate in a pilot has been suggested before,^10,11^ there is a lack of methodological guidance for determining exactly how much it should be relaxed by, or for choosing an appropriate pilot sample size.

One possible approach to defining optimal error rates is through Bayesian statistical decision theory. Under this framework we define a suitable utility function which encodes our preferences, and make decisions based on the expected value of this utility with respect to a prior distribution which expresses our uncertainty on the unknown parameters. Although the theory is well established^12–14^ and has been proposed in previous methodological work around optimal trial design,^15^ it has been argued that the requirement of specifying a utility function has led to low uptake in practice.^16^

In this article, we aim to propose a simple and general form for a utility function in two-arm, randomised, parallel group clinical trials, making clear the assumptions which are encoded in it and thus allowing its applicability or otherwise to the problem at hand to be judged. The utility we propose is closely related to several existing proposals in the literature,^17^ but with some key differences. One particular aspect we have considered is the decision-maker’s attitude to risk, an issue sidestepped by many existing proposals which assume, explicitly or implicitly, that the decision-maker is risk-neutral. We will show that the attitude to risk can have a considerable influence on optimal trial design, and is key to answering the principle motivating question of this paper: in what situations, if any, is it optimal to *not* test effectiveness in a pilot trial?

The remainder of this article is structured as follows. We define the specific problem under consideration in Section 2, and describe the proposed method in Section 3. In Section 4, we illustrate the application of the method to design an external pilot of a complex intervention. We evaluate the properties of the method over a range of possible scenarios in Section 5, and then outline some extensions in Section 6. Finally, we conclude with a discussion of the strengths and limitations of the proposed approach in Section 7.

## 2. Problem

Consider the problem of jointly designing an external pilot trial and subsequent definitive trial. We will denote these, respectively, as stages i=1 and i=2 of the overall programme. We consider the case where both trials are parallel group studies comparing an intervention to control. We assume that the comparison focuses on superiority in terms of the mean difference of a normally distributed primary endpoint with known standard deviation. For simplicity, we also assume that this standard deviation is common to both arms, although our approach can be applied equally to the heteroskedastic case. Finally, we assume that the endpoint is identically distributed within arms in both the pilot and definitive trial.

We denote the true mean difference by μ , and consider the case where the primary analysis at each stage will be a z-test of the null hypothesis $H_{0}:\mu =0$ . The test at stage i will compare the sample mean difference between groups, denoted $x_{i}$ , to a pre-specified critical value, denoted $c_{i}$ . At the pilot stage, a positive result (i.e. $x_{1}>c_{1}$ ) will indicate that we should proceed to the definitive trial. At the definitive stage, a positive result (i.e. $x_{2}>c_{2}$ ) will indicate that the intervention should be recommended for use over the control treatment. The thresholds $c_{1},c_{2}$ , along with the per-arm sample sizes at each stage $n_{1},n_{2}$ , collectively define the design of the overall programme. The problem we consider in this article is to optimise $n_{i},c_{i},i=1,2$ .

Given some alternative hypothesis $H_{1}:\mu =\mu^{*}$ , we define the following operating characteristics:

$$\begin{array}{rl} \alpha_{i} & =Pr[x_{i}>c_{i}|\mu =0]\\ \beta_{i} & =Pr[x_{i}\leq c_{i}|\mu =\mu^{*}] \end{array}$$

These represent the type I and II error rates of the tests performed at each stage i=1,2 , and provide an alternative summary of the pilot and definitive trial programme. From these we can also derive the overall type I and II error rates of the programme. The probability of obtaining a final statistically significant result under the null hypothesis is $\alpha_{t}=\alpha_{1}\alpha_{2}$ , since the events of obtaining significant results in stages i=1 and i=2 are independent. Similarly, the overall probability of failing to observe a final statistically significant result under the alternative hypothesis is $\beta_{t}=\beta_{1}+(1-\beta_{1})\beta_{2}$ .

## 3. Maximising expected utility in trial programmes

We consider a Bayesian view of the frequentist design problem, and, therefore, require a prior distribution for the unknown true mean difference μ . This prior information will be used only to guide the choice of the frequentist design and analysis parameters, and not in any analysis of the trial data itself. As such, a non- or weakly informative prior is not appropriate; rather, the prior should be a subjective summary of the decision-maker’s knowledge and uncertainty about μ . For computational tractability, we will assume a normal prior p(μ) with mean m and variance $s^{2}$ .

We define optimal design variables as those which maximise the expectation, with respect to the prior p(μ) , of a utility function. We construct the utility function in three steps, following the procedures described by Keeney and Raiffa.^13^ First, we identify the *attributes* which we consider will be of interest to the decision maker. We propose these are the total sample size of the trial programme, n , the change in mean outcome following the trial programme, d . and b , an indicator where b=0 if the experimental treatment is adopted and b=1 otherwise.

We then define a *value function* over the space of these attributes, which encodes the decision-maker’s preferences under conditions of certainty. We propose that this takes the form of a weighted sum of the three attributes, denoting the weights by $k_{n},k_{d}$ and $k_{b}$ . This gives values of $k_{b}$ for retaining the control treatment and $k_{d}d$ for adopting the experimental treatment, indicating the latter will be preferred for sufficiently large d . These values are then set against the cost of sampling, $k_{n}n$ . The weights can be determined by eliciting two quantities: $\bar{d}$ , a change in mean outcome that would justify increasing the total sample size from 0 to $n_{*}$ ; and $\hat{d}$ , a change in mean outcome that would justify switching from the current standard treatment to the intervention under study. Having elicited these, we have

(1) $$k_{n}=-k_{d}\bar{d}/n_{*},\;k_{d}=1/(1+\hat{d}-\bar{d}/n_{*}),\;k_{b}=1-k_{d}-k_{n}\hat{d}$$

We then transform the value function into a *utility function* by incorporating the decision-maker’s attitude to risk. Drawing on Bayesian decision theory,^13^ we find that the structure of the value function implies the utility function must be of the form

(2) $$u(n,d,s)=\left\{ \begin{array}{ll} 1-e^{-\rho (k_{n}n+k_{d}d+k_{b}b)}, & \rho >0\\ k_{n}n+k_{d}d+k_{b}b, & \rho =0\\ -1+e^{-\rho (k_{n}n+k_{d}d+k_{b}b)}, & \rho <0 \end{array}\right.$$

where the parameter ρ represents the decision maker’s attitude to risk with respect to uncertainty in the overall value of the three attributes. Here, ρ>0 implies risk aversion, ρ=0 risk neutrality, and ρ<0 a risk-seeking attitude. Full details of the derivation of equation (2) and suggestions of how the parameters $\bar{d},\hat{d}$ and ρ can be elicited are given in the appendix.

### 3.1. Expected utility

Denote by $G_{i}$ an indicator variable where $G_{i}=1$ if there is a positive test result at stage i , and $G_{i}=0$ otherwise. For the problem considered here, $G_{i}=1\Leftrightarrow x_{i}>c_{i}$ . Noting that the attributes d , n and b are completely determined by the fixed programme design $z=(n_{1},c_{1},n_{2},c_{2})$ , the realisations of $G_{1}$ and $G_{2}$ , and the true treatment effect μ , we re-write utility as $u(\mu ,G_{1},G_{2}\;|\;z)$ . Focusing on the case where ρ>0 (the other cases will follow), we have

(3) $$\begin{array}{rl} u(\mu ,G_{1},G_{2}\;|\;z) & =1-\exp\;(-\rho [k_{d}\mu +k_{n}(n_{1}+n_{2})]G_{1}G_{2}\\ & \;-\rho [k_{n}(n_{1}+n_{2})+k_{b}]G_{1}(1-G_{2})\\ & \;-\rho [k_{n}n_{1}+k_{b}](1-G_{1})) \end{array}$$

The expected utility conditional on μ is

(4) $$\begin{array}{rl} E[u(\mu ,G_{1},G_{2}\;|\;z,\mu )] & =Pr[G_{1}=1,G_{2}=1|z,\mu ]\left(1-e^{\rho (k_{d}\mu +k_{n}(n_{1}+n_{2})}\right)\\ & \;+Pr[G_{1}=1,G_{2}=0|z,\mu ]\left(1-e^{-\rho (k_{n}(n_{1}+n_{2})+k_{b})}\right)\\ & \;+Pr[G_{1}=0|z,\mu ]\left(1-e^{-\rho (k_{n}n_{1}+k_{b})}\right) \end{array}$$

Since the sample means are conditionally independent and normally distributed as $x_{i}|\mu ∼N(\mu ,2\sigma^{2}/n_{i})$ , the conditional probabilities in equation (4) are easily calculated. We are then left with integrating out the unknown treatment effect μ :

(5) $$E[u(\mu ,G_{1},G_{2}\;|\;z)]=\int E[u(\mu ,G_{1},G_{2}\;|\;z,\mu )]p(\mu )d\mu$$

As we are integrating with a normal density weighting function, we can use Gauss-Hermite quadrature (implemented in the ‘fastGHQuad‘ R package^18^) to evaluate this integral.

### 3.2. Optimisation

Optimal programme designs can be found by solving the optimisation problem

(6) $$\begin{array}{rl} \max_{z=(n_{1},c_{1},n_{2},c_{2})} & E[u(\mu ,G_{1},G_{2}\;|\;z)]\\ \text{s.t.} & n_{i}\in N,i=1,2\\ & c_{i}\in R,i=1,2 \end{array}$$

for a given prior distribution for the unknown μ . To solve this problem, we use the gradient-assisted local optimisation method of Byrd et al.^19^ as implemented in the R^20^ function ‘optim’. Full details are provided in the Supplemental Material.

## 4. Illustration

OK-Diabetes aimed to assess the feasibility of evaluating supported self-management for adults with learning disabilities and type II diabetes.^21^ The original target sample size was 30 patients per arm, chosen based on a rule-of-thumb^5^ and to allow the feasibility objectives of the study to be addressed. The team were asked by the funder to consider assessing the potential efficacy of the intervention to determine whether a confirmatory trial should go ahead. A continuous measure of the percentage difference in participant blood sugar levels (HbA1c) from baseline to six months was chosen as the efficacy outcome. The standard deviation of this outcome was identified to be 1.5%.^22^ A mean change of 0% was considered to be of no interest, whilst a mean reduction of 0.5% at 6 months was deemed the target difference.

The target sample size was increased to 56 participants per arm, giving $1-\beta_{1}=0.82$ power to detect a true mean reduction of 0.5% using a one-sided test with a type I error rate of $\alpha_{1}=0.2$ . Although the error rates for the subsequent definitive trial were not specified, we note that a sample size of 190 participants per arm would lead to $1-\beta_{2}=0.9$ power to detect a true mean reduction of 0.5% using a conventional one-sided type I error rate of $\alpha_{2}=0.025$ . In this section, we consider how the proposed method could be used to determine optimal choice of $z=(n_{1},c_{1},n_{2},c_{2})$ or, equivalently (see Section 2), of the operating characteristics $\alpha_{i},\beta_{i},i=1,2$ .

### 4.1. Prior and utility

To apply the proposed method, we require a prior distribution on the treatment difference p(μ) and a utility function u(.) . For the former, we use a conjugate normal prior with parameters m=0 and s=0.6 . This represents a sceptical prior, being centred at the null hypothesis of no difference and with a variance corresponding to a prior belief that μ≥0.5 with a probability of ∼ 0.20.

For the utility function, we first consider the change in outcome which would be enough to justify the costs of switching from the current standard treatment to the new treatment under study. To determine this value we note that a conventional definitive trial design, with a type I error rate of 0.025, the sample size of 191 participants per arm and a power of 0.9 to detect μ=0.5 , would lead to 0.5 power when μ≈0.3 . This implies an indifference between adopting the new treatment and staying with the current standard if this was the true treatment difference,^23^ and thus gives a rationale for choosing $\hat{d}=0.3$ . For the cost of sampling, we seek to identify a change in treatment effect which would justify an increase in the sample size from 0 to $n_{*}=50$ (where the choice of $n_{*}$ is arbitrary). For the purposes of illustration, we suppose that this leads to $\bar{d}=0.005$ , meaning that we consider an increase in sample size of 5000 to be worth paying if we obtained a *guaranteed* change in treatment effect of 0.5, the target difference in this problem.

Given these judgements and using equation (1), we have the value function

v(n,d,b)=0.769d−0.0000769n+0.231b

Moving to utility, we set $d_{\min}=0$ and $d_{\max}=0.5$ (arbitrarily) and consider the change of treatment we would like to obtain for certain for it to be judged equivalent to a simple 50/50 gamble between $d_{\min}$ and $d_{\max}$ . We suppose a risk-averse attitude leads to a choice of 0.19 , corresponding to ρ=2 . Our utility function is then

u(n,d,b)=1−exp[−2×(0.769d−0.0000769n+0.231b)]

### 4.2. Optimal design

We consider two variations of the optimal design problem. First, we optimise jointly over the pilot and main trial programme (‘unrestricted’). Then, we optimise only the main trial whilst fixing $\alpha_{1}=1,\beta_{1}=0$ (‘no pilot test’). In both cases, we note that the original OK-Diabetes sample size of 30 per arm was intended to allow feasibility questions to be addressed, and so we set this as a lower limit of $n_{1}$ (we will explore the effect of removing this lower limit in Section 5). The algorithm takes around 1 second to converge to a solution. The results are given in Table 1.

**Table 1.** Optimal sample size and error rates for the OK-Diabetes external pilots trial ( i=1 ) and subsequent definitive trial ( i=2 ), for the general unrestricted case and where we insist on not testing effectiveness in the pilot trial.

Problem	$n_{1}$	$n_{2}$	$\alpha_{1}$	$\beta_{1}$	$\alpha_{2}$	$\beta_{2}$	Expected utility
Unrestricted	41	146	0.39	0.110	0.041	0.132	−0.42874
No pilot test	30	110	1.00	0.000	0.036	0.254	−0.42292

In the unrestricted case we find that the optimal programme involves an external pilot sample size of $n_{1}=41$ participants per arm, between the initial and revised choices of sample size of 30 and 56 used in OK-Diabetes. The balance of error rates in the pilot is, however, substantially different to those chosen previously. We find that a large stage-1 type I error rate of $\alpha_{1}=0.39$ (one sided) is used, allowing a high power of $1-\beta_{1}=0.89$ whilst maintaining a low sample size. Having allowed a large type I error rate in the pilot, the optimal definitive trial uses a lower stage-2 type I error $\alpha_{2}=0.041$ . In isolation this is somewhat higher than the conventional choice of 0.025, but note that when combined with the type I error rate of the pilot trial it leads to an overall type I error rate of $\alpha_{t}=0.016$ . The optimal definitive sample size of 146 per arm then corresponds to a power of 0.868, with an overall power for the programme of $1-\beta_{t}=0.773$ .

When we insist on not testing in the external pilot we obtain a lower definitive trial sample size of $n_{2}=110$ , with type I error rate $\alpha_{2}=0.036$ and power $1-\beta_{2}=0.746$ . The expected utility of this programme is 0.00582 lower than the optimal unrestricted programme. To interpret this, we can translate utilities back to values and then into attribute units. Specifically, note that the utility function implies that an expected utility of x can be translated into a value of

$$-\frac{1}{\rho }\ln\;(1-x)$$

A difference in utilities $x_{1}-x_{2}$ can, therefore, be translated into a difference in values, and this can then be divided by $k_{n}$ to put it in units of sample size:

(7) $$\frac{1}{\rho k_{n}}\left[\ln\;(1-x_{2})-\ln\;(1-x_{1})\right]$$

For the ρ=2 in our example the two optimal solutions have values of 0.2800 and 0.2749 , giving a difference in value of 0.0051 . Dividing this by $k_{n}=-0.0000769$ leads to an effective difference of 66 participants. That is, we can consider the unrestricted optimal design to be more efficient than the restricted design by an amount equivalent to recruiting and following up 66 participants. Thus, in this case, the conventional policy of not testing effectiveness in pilot trials is considerably inefficient.

To examine the effect of the pilot sample size on the expected utility of the programme, we varied $n_{1}$ in the range [30,56] and, optimising over the remaining parameters, calculated the improvement over the ‘no pilot test‘ approach in units of sample size. The lowest improvement in this range was ∼ 64 participants, indicating that the benefits derived from the ‘unrestricted‘ approach stem principally from the ability to test effectiveness at the pilot stage, as opposed to any particular choice of pilot sample size.

### 4.3. Sensitivity analysis

The suggested programme design is optimal only for a certain choice of prior and utility parameters, and so it is of interest to assess how robust the design is to deviations from these. To do this we consider a range of alternative parameter values and, for each, determine the optimal programme design. The expected utility of this optimal design can then be compared against that of the proposed design, converted into units of sample size as above in equation (7). We will refer to this difference as the *regret*. For example, the regret associated with the ‘no pilot test’ approach in Table 1 was 66 participants. We conducted two sensitivity analyses: first, we varied the prior parameters m and s ; secondly, we varied the utility parameters ρ and $\bar{d}$ . All other parameters were kept at their original values.

**Figure 2.**
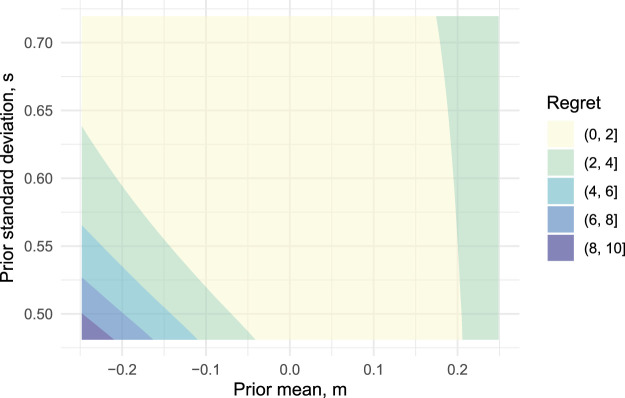
Amount of regret when using the proposed OK-Diabetes programme design as the prior mean m and prior standard deviation s vary. The boundaries of the shaded areas are contours with regret values of 2,…, 10.

Figure 2 plots the regret over a range of prior means m and prior standard deviations s . We varied the prior mean from − 0.5 to 0.5, moving from extremely sceptical to enthusiastic beliefs. We find that over this range there is little to be gained from moving from the proposed design to the locally optimal design, providing the prior standard deviation is equal to or greater than the initial choice of s=0.6 . As we decrease s down to 0.48 the penalty of using the proposed design can increase, but the magnitude of these penalties depends on m . From these results, we can conclude that the proposed design is quite robust to misspecification of the prior distribution, in the sense that if the choices of m,s are not quite an accurate reflection of our prior beliefs, the design will still have an expected utility close to that of the true optimal design.

**Figure 3.**
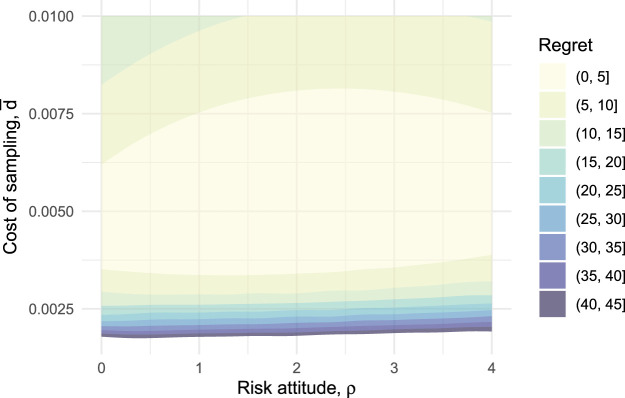
Amount of regret when using the proposed OK-Diabetes programme design as the attitude to risk ρ and cost of sampling $\bar{d}$ vary. The boundaries of the shaded areas are contours with regret values of 5, 10,…,45.

Corresponding results for varying utility parameters ρ and $\bar{d}$ are given in Figure 3. We see that the proposed design is quite robust to misspecification of the attitude to risk, and to underestimation of the cost of sampling. However, if the cost of sampling is initially overestimated, the proposed design can become considerably sub-optimal. For example, maintaining ρ=2 but halving the cost of sampling from 0.005 to $\bar{d}=0.0025$ means the proposed design is worse than the true optimal design by an amount equivalent (through application of equation (7) to 24 participants. This analysis suggests that the choice of $\bar{d}$ , in particular, should be carefully examined to ensure it is a true reflection of the decision-maker’s preferences.

## 5. Evaluation

In the OK-Diabetes example, we found that the standard policy of not testing for efficacy in an external pilot trial can be considerably sub-optimal. Here, we consider a range of different utility function parameter values and examine when, if at all, not testing in the pilot trial is optimal. Throughout, we maintain the same sceptical prior with m=0 and s=0.6 . We considered the nine scenarios formed by setting the cost of sampling $\bar{d}$ to one of {0.0025,0.005,0.01} , and the treatment cost parameter $\hat{d}$ to one of {0.1,0.2,0.3} . For each of the nine scenarios, we varied the attitude to risk, with ρ∈[−5,5] , finding optimal programme designs over this range. We did this for two cases: firstly, assuming that a pilot sample size of $n_{1}\geq 30$ is required in order to address feasibility questions; and secondly, removing this lower bound.

### 5.1. The case $n_{1}\geq 30$

**Figure 4.**
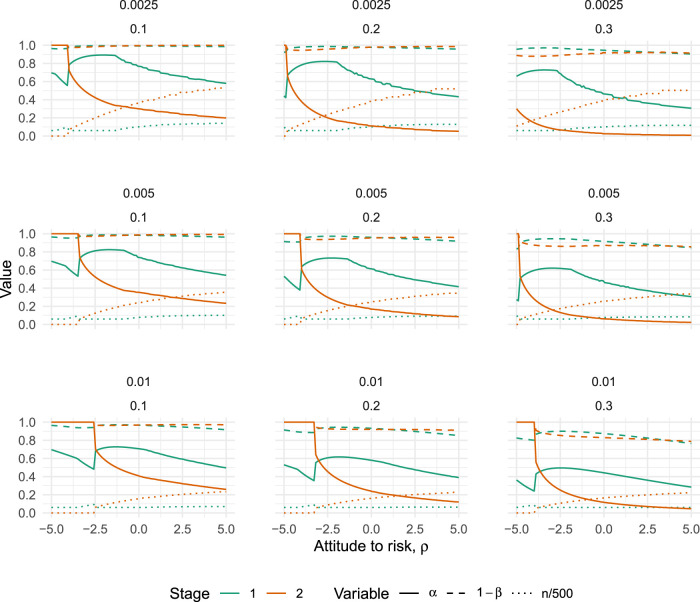
Optimal type I error rates (solid lines), type II error rates (dashed lines) and scaled sample size (dotted lines) for varying values of ρ (the attitude to risk, where higher means more risk-averse), when the pilot sample size is constrained to $n_{1}\geq 30$ . Plots vary horizontally with treatment costs, $\hat{d}\in \{0.1,0.2,0.3\}$ , and vertically with sampling costs, $\bar{d}\in \{0.0025,0.005,0.01\}$ .

The results are given in Figure 4, which plots how the error rates of both the pilot ( i=1 ) and definitive ( i=2 ) trials vary with ρ for each of the nine scenarios. It is always optimal to test for effectiveness in the pilot trial in these scenarios, although the type I error rate used can be quite high. The largest we found was $\alpha_{1}=0.89$ , when $\rho =-1.8,\bar{d}=0.0025$ and $\hat{d}=0.1$ (top left panel in Figure 4). The trends in Figure 4 suggest that decreasing $\bar{d}$ and/or $\hat{d}$ could potentially lead to higher $\alpha_{1}$ , but we failed to find any case where $\alpha_{1}=1$ .

The broad trends which emerge from Figure 4 are that optimal type I errors tend to decrease as we become more risk-averse, while optimal type II errors stay relatively stable. As the treatment costs increase (moving from left to right in Figure 4), both type I and II errors tend to decrease. And, as the cost of sampling increases (moving from top to bottom in Figure 4), both type I and II errors tend to decrease. In all nine scenarios, we find there is a point where the definitive trial jumps to an optimal design of $n_{2}=0,\alpha_{2}=1,1-\beta_{2}=1$ , meaning the pilot trial is the only trial which will be run. The point where this happens is always for a negative value of ρ . That is, there is a point where a sufficiently risk-seeking attitude will imply the optimal action is to run only one trial.

### 5.2. The case $n_{1}\geq 0$

**Figure 5.**
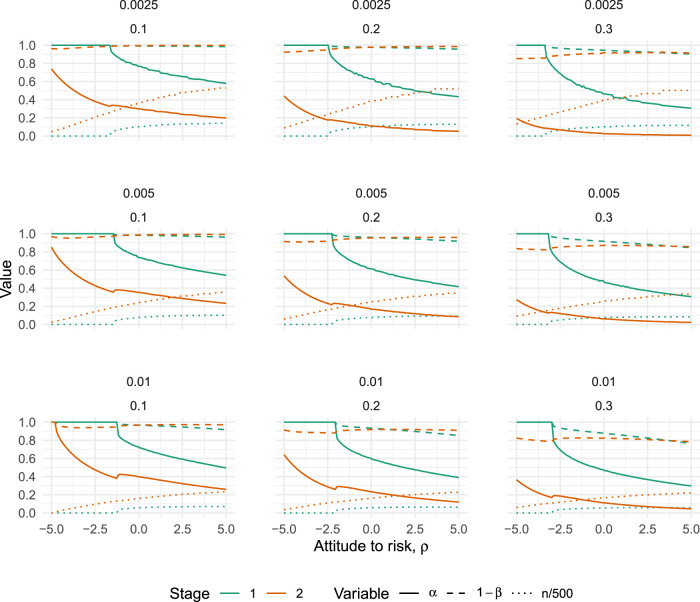
Optimal type I error rates (solid lines), type II error rates (dashed lines) and scaled sample size (dotted lines) for varying values of ρ (the attitude to risk, where higher means more risk-averse), when the pilot sample size is unconstrained. Plots vary horizontally with treatment costs, $\hat{d}\in \{0.1,0.2,0.3\}$ , and vertically with sampling costs, $\bar{d}\in \{0.0025,0.005,0.01\}$ .

We now examine the characteristics of optimal programmes with no lower bound on the sample size at the pilot stage. This will be the case when the purpose of the pilot trial is only to assess effectiveness, as opposed to feasibility, and is similar to the problems considered in related work on optimal pilot and phase II trial design.^24,25^ The results are given in Figure 5, which plots how the error rates of both the pilot ( i=1 ) and definitive ( i=2 ) trials vary with ρ , for each of the nine scenarios.

The trends of how optimal error rates and sample sizes vary with the utility function parameters are broadly similar to those shown in Figure 4. We see similar inflection points, where now a sufficiently risk-seeking attitude will result in an optimal pilot trial sample size of $n_{1}=0$ , leaving only the definitive trial to be conducted. Optimal pilot trial type I error rates are only found to be $\alpha_{1}=1$ when $n_{1}=0$ . In the scenarios considered here, we again fail to find a situation where it is optimal to run a pilot trial but not test for effectiveness.

## 6. Extensions

### 6.1. Internal pilots

Internal pilot trials are distinguished from external pilots by their data being used at the final analysis, with a seamless gap between the pilot and definitive trial stages. Extending our problem to the internal pilot setting, we continue to conduct a first test based on the pilot sample mean difference $x_{1}$ , but now follow this with a test of the overall sample mean difference $x_{t}$ , where

$$x_{t}=\frac{n_{1}x_{1}}{n_{1}+n_{2}}+\frac{n_{2}x_{2}}{n_{1}+n_{2}}$$

We can now apply equation (4) in the internal pilot by defining $G_{1}=x_{1}>c_{1}$ and $G_{2}=x_{t}>c_{2}$ . The relevant probabilities can be calculated by noting that the pair $x_{1},x_{t}$ , conditional on μ , follow a bivariate normal distribution. Specifically (see the Appendix),

$$\left(\begin{array}{c} x_{1}\\ x_{t} \end{array}\right)|\mu ∼N\left(\left(\begin{array}{c} \mu \\ \mu \end{array}\right),\left(\begin{array}{cc} \frac{2\sigma^{2}}{n_{1}} & \frac{2\sigma^{2}}{n_{1}+n_{2}}\\ \frac{2\sigma^{2}}{n_{1}+n_{2}} & \frac{2\sigma^{2}}{n_{1}+n_{2}} \end{array}\right)\right)$$

The probabilities in equation (4) are now with respect to this bivariate normal distribution, and can be calculated using (for example) the R package ‘mvtnorm’.^26^ Expected utility can then be calculated as before, integrating the conditional expected utility over the normal prior p(μ) using quadrature.

The optimal internal pilot and definitive trial programme for the OK-Diabetes example is given in Table 2, where we also include the optimal programme for the external pilot case as found in Section 4. We find that the overall type I error rates are approximately equal for both the external and internal pilot cases, and overall type II rates are very similar. The internal pilot programme has a slightly higher expected utility, which we might expect given the fact that all of the data is being utilised in the final analysis.

**Table 2.** Optimal sample size and error rates for the OK-Diabetes pilot trial ( i=1 ) and subsequent definitive trial( i=2 ), when the pilot is external and internal.

Problem	$n_{1}$	$n_{2}$	$\alpha_{1}$	$\beta_{1}$	$\alpha_{t}$	$\beta_{t}$	Expected utility
External	41	146	0.39	0.110	0.016	0.228	−0.42874
Internal	45	121	0.42	0.084	0.016	0.213	−0.42954

### 6.2. Heterogeneous effects

We have assumed to this point that the treatment effect μ is the same at both the pilot and main trial stages, but now relax this assumption to allow the effect in pilot trial, $\mu_{p}$ , to differ, thus leading to the type of bias highlighted by Sim.^8^ Specifically, we model the effect vector using the bivariate normal prior distribution

$$\left(\begin{array}{c} \mu_{p}\\ \mu \end{array}\right)∼N\left(\left(\begin{array}{c} m_{p}\\ m \end{array}\right),\left(\begin{array}{cc} s_{p}^{2} & \tau s_{p}s\\ \tau s_{p}s & s^{2} \end{array}\right)\right)$$

Calculating expected utility proceeds largely as before, but now the probabilities in equation (4) are based on the distribution of the pilot estimate $x_{1}$ conditional on the true main trial effect μ :

$$x_{1}\;|\;\mu ∼N\left(m_{p}+\tau \frac{s_{p}}{s}(\mu -m),(1-\tau^{2})s_{p}^{2}+\frac{2\sigma^{2}}{n_{1}}\right)$$

For example, in the OK-Diabetes example we suppose that the pilot effect has the same marginal mean and standard deviation as the definitive trial effect (i.e. $m_{p}=0$ and $s_{p}=0.6$ ). Suppose further that we set the prior correlation between the true pilot and definitive trial effects to be τ=0.9 , noting that this is a relatively weak correlation in our context; it implies that our prior belief regarding the main trial effect μ would have a standard deviation of 0.26 even if the true pilot trial effect $\mu_{p}$ was known. Given this joint prior distribution, the optimal programme is given in Table 3. We provide the optimal programme in the case of perfect correlation for comparison.

**Table 3.** Optimal sample size and error rates for the OK-Diabetes external pilot trial ( i=1 ) and subsequent definitive trial ( i=2 ), for different correlations between pilot and main trial effects τ .

τ	$n_{1}$	$n_{2}$	$\alpha_{1}$	$\beta_{1}$	$\alpha_{2}$	$\beta_{2}$	Expected utility
0.9	30	134	0.69	0.963	0.034	0.818	−0.42656
1.0	41	146	0.39	0.890	0.041	0.868	−0.42874

**Figure 6.**
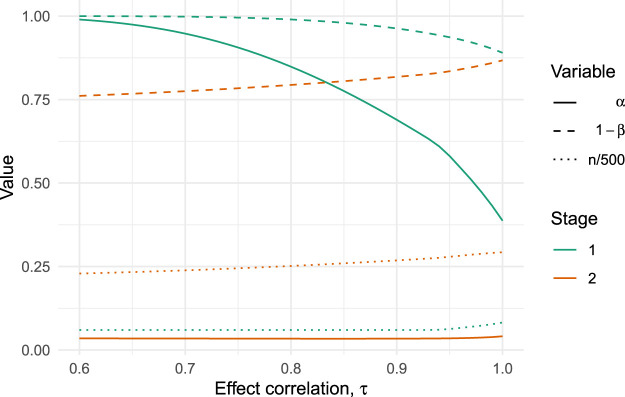
Optimal type I error rates (solid lines), type II error rates (dashed lines) and scaled sample size (dotted lines) for varying values of τ (the correlation between pilot and main trial effects) in the OK-Diabetes example.

As we might expect, a less-than-perfect correlation reduces the optimal sample size of the pilot trial and increases its optimal type I error rate. This trend continues as we further reduce τ , as shown in Figure 6. We find that τ must be as low as 0.6 for the value of testing effectiveness in the pilot to diminish and the optimal type I error rate approach 1. Repeating this analysis for different values of ρ , the attitude to risk, shows that the point at which the optimal pilot type I error rate approaches 1 increases as ρ decreases and we become more risk-seeking (results not shown here, but see the supplementary material for the required code).

## 7. Discussion

We have explored how Bayesian statistical decision theory can be used to define optimal type I and II error rates for trial programmes involving a pilot trial and a subsequent definitive trial. We have introduced a general utility function, outlining the associated assumptions, and demonstrated how its parameter values can be determined. When evaluating the conventional approach to pilot trial analysis we found that a policy of not testing effectiveness was consistently sub-optimal, even when we allowed for heterogeneity between the effects at the pilot and main trial stages. As a result, we recommend that pilot data can and should be used to conduct a preliminary test of effectiveness prior to the definitive trial, when the assumptions around the data generating mechanism, prior distributions and utility function described in this article hold. This would lead to a considerable improvement in the complex intervention evaluation pathway, as more ineffective interventions are identified and screened out at the pilot stage.

A key component of the decision-theoretic approach is the utility function. For simplicity, we did not include any set-up costs relating to the pilot or definitive trial. If these are important, expressing them in units of sample size would allow them to be included in the model easily. In terms of the resulting effect on optimal design characteristics, set-up costs would mean a design with either $n_{1}=0$ or $n_{2}=0$ becoming more attractive. As such, we might expect to see such designs becoming optimal over a larger range of values for ρ in Figures 4 and 5. We did not attempt to predict the number of patients who will be affected by the results of the definitive trial, or the manner in which they will adopt the intervention following a significant result. Were such a model to be included, the utility function could be re-expressed in terms of individual patient outcomes rather than population parameters, allowing the utilities of the people participating in the trial to be weighted equally against the utilities of those who stand to benefit from the trial results. Such considerations will be particularly important in small population contexts, such as with rare diseases, where the trial population can form a considerable fraction of the overall target population.^17^ The exponential form of the utility function was derived from an additive value function and an assumption of utility independence, in addition to an assumed mutual preferential independence between the three attributes. Although the appropriateness of these assumptions must be judged in light of the problem at hand, we note that an additive utility function is often assumed in related decision-theoretic work.^17,23,27–29^ As shown in equation (2), an additive utility entails these assumptions while also assuming risk-neutrality on the part of the decision maker. Our approach can, therefore, recover risk-neutrality as a special case, while also being flexible enough to accommodate risk-averse and risk-seeking attitudes (noting that we would not generally expect to see the latter in the context of our trial design problems). We also emphasise that the utility parameters used in this paper are hypothetical. Future work could examine how the elicitation procedures described in the Appendix work in practice to help understand the feasibility of the proposed approach.

We have considered programmes where a hypothesis test is used in the primary analysis of the pilot and definitive trials. The type I error rates of the suggested optimal programmes have not been restricted, but if this is desired (e.g. the overall type I error rate $\alpha_{t}$ may need to be < 0.025 for regulatory purposes) the optimisation problem (6) could be augmented by adding appropriate constraints.^30^ Further work could explore how a Bayesian analysis of pilot trial data could be used to update prior beliefs and use the revised knowledge to optimise the subsequent definitive trial. At the programme design stage, the pilot trial sample size could then be determined using value of information methods.^23^ A potential difficulty with such an approach is the computational aspect of such calculations, although techniques for enabling fast calculation of the expected value of sample information may be useful in this context.^31,32^

We have focused on using pilot trials to test the efficacy of the intervention, but the broad strategy outlined here is quite flexible and could be applied or extended to other settings. For example, it could be used to optimise the design of a single confirmatory trial, helping us find the optimal balance of error rates and sample size.^33,34^ Programmes of non-inferiority trials could be considered by allowing for negative choices of the parameter $\hat{d}$ , which denotes the amount of treatment difference we would consider equivalent to the costs of adopting the new treatment. The assumption of known variance could easily be relaxed by using *t*-tests when calculating the probabilities of equation (4) and integrating over a joint prior of effect and outcome variance. When we also want to allow for unequal variance in the two arms of the trial, we can apply the Satterthwaite approximation^35^ to the degrees of freedom of the *t*-test, and integrate over a bivariate prior of the two components of the outcome variance. The method for internal pilots described in Section 6.1 could be further extended to the general group sequential setting by allowing for more than one interim analysis and including an option to stop for efficacy as well as for futility. A more involved extension would be to recognise that pilot trials are often used to estimate other parameters relating to the feasibility of the definitive trial, such as recruitment, follow-up and adherence rates.^36^ These parameters have clear implications for the duration, cost and value of a trial, and as such could be included in the utility function so that learning about them can be offset against the cost of sampling.

The optimisation problem stated in Section 3.2 is not trivial, and we found some variability in performance of different optimisation algorithms. The suggested method was found to be robust, but it would be advisable to check for global convergence when applying to a given problem. This could be done by using other algorithms, such as the genetic optimisation algorithms implemented in the ‘rgenoud’ package,^37^ to check they agree or by using different starting points. Alternatively, several closely related problems could be solved and the resulting optimal programme characteristics plotted, much as we have done in the sensitivity analyses of Section 4.3. We would expect to see smooth variation, with any erratic behaviour would suggest some convergence issues. Note this is exemplified in Figure 5, where some small blips in the operating characteristic curves can be seen and would suggest a slight failure in convergence at these points. Alternative optimisation approaches may help to address these problems. For example, we could use exhaustive or bisection searches over the sample sizes $n_{1}$ and $n_{2}$ , solving the simpler problem of optimising the critical values in each case. As noted in Section 3, the use of a normal prior for the treatment effect aids computational tractability. If an alternative prior is deemed appropriate then the numerical integration in equation (5) would require more general quadrature or Monte Carlo methods, which will increase the time required to solve the optimisation problem.

The majority of our work has assumed the effect sizes in the pilot and definitive trials are equal, which we then relaxed in Section 6 by using a joint prior distribution for the two effects which allows for a correlation of τ<1 . When applied to our illustrative example we found that testing effectiveness in the pilot remains optimal for τ≥0.6 , with considerable benefits when τ≥0.9 . As noted in Section 6.2, this is a relatively weak correlation which implies that the marginal standard deviation for the definitive effect prior reduced from 0.6 to only 0.26 when conditioning on the true pilot effect. Empirical studies comparing pilot and definitive trial pairs could potentially provide information to inform these prior beliefs.^38^ Our results suggest that there is value in trying to minimise the differences between the pilot and definitive trial effects. One way to do that would be to avoid the common practice of making modifications to the intervention following the pilot trial in an attempt to improve it, potentially by instead approaching the question of intervention optimisation through the Multiphase Optimisation Strategy (MOST).^39^

## Supplemental Material

sj-pdf-1-smm-10.1177_09622802251322987 - Supplemental material for Optimising error rates in programmes of pilot and definitive trials using Bayesian statistical decision theory Supplemental material, sj-pdf-1-smm-10.1177_09622802251322987 for Optimising error rates in programmes of pilot and definitive trials using Bayesian statistical decision theory by Duncan T Wilson, Andrew Hall, Julia M Brown and Rebecca EA Walwyn in Statistical Methods in Medical Research

## Appendix

### Defining the utility function

#### Attributes

The first attribute of interest is the change in average outcome for the patient population, in terms of the primary endpoint, after the trial programme has been conducted. We denote this change by d . It will be determined by three factors: the trial outcomes (in terms of the binary hypotheses test results); the true treatment difference μ ; and the manner in which patients adopt the new treatment if the definitive trial concludes it is effective. Considering the latter requires a model predicting how patients will choose treatments. For simplicity, we will use a simple model assuming that the whole population will adopt the new treatment if the result of the definitive trial is positive, leading to a change in outcome of d=μ . Otherwise, the population will retain the control treatment and the change in outcome will be d=0 .

The sample size at both stages of the programme is of interest, being associated with both monetary costs and the exposure of patients to research. The total sample size will vary from programme to programme, both by design and through the uncertain outcome of the pilot trial. Assuming that sampling at each stage is equivalent in terms of the costs involved, we can then identify the total sample size $n=n_{1}+n_{2}$ as our second attribute.

While the focus of comparison between the intervention and control is in terms of the primary outcome, the treatments will in general differ in other aspects. We limit ourselves to the case where these differences are deterministic and known, and will refer to them as the treatment costs associated with the intervention. In this case, we can encapsulate these differences in a single indicator variable, and our third attribute, b∈{0,1} , where b=1 if and only if we decide to retain the control treatment.

#### Value

Considering preferences under conditions of certainty, we use the notation $y≺y^{\prime }$ to mean y is preferred to $y^{\prime }$ , and $y∼y^{\prime }$ to mean indifference between y and $y^{\prime }$ . We assume that ≺ is a weak ordering (i.e. complete and transitive) of all possible values y . We aim to specify a value function v(n,d,b) which will assign to each possible set of attribute values a real number (its *value*) which corresponds with the qualitative preferences of the decision maker. That is, we require v(n,d,b) such that

$$\left(n,d,b)≺(n^{\prime },d^{\prime },b^{\prime })\Leftrightarrow v(n,d,b)<v(n^{\prime },d^{\prime },b^{\prime }\right)$$

for all $\left(n,d,b),(n^{\prime },d^{\prime },b^{\prime }\right)$ . We will use a value function with the following additive form:

$$v(n,d,b)=v_{n}(n)+v_{d}(d)+v_{b}(b)$$

where $v_{n},v_{d}$ and $v_{b}$ are value functions representing preferences over each of the attributes when considered in isolation. It has been shown that such an additive value function exists if and only if each pair of attributes is *preferentially independent* of the remaining attribute.

Definition (Preferential independence (Keeney and Raiffa,^13^ p. 101)) The pair of attributes X and Y is preferentially independent of attribute Z if $\left(x^{\prime },y^{\prime },z)≺(x^{\prime \prime },y^{\prime \prime },z)⟺(x^{\prime },y^{\prime },z^{\prime })≺(x^{\prime \prime },y^{\prime \prime },z^{\prime }\right)$ for any $z,z^{\prime }$ .

This condition requires, for example, that attributes n and b are preferentially independent of attribute d in the sense that preferences on the (n,b) space for a fixed $d^{\prime }$ do not depend on the specific value of $d^{\prime }$ .

We impose further structure on the value function by assuming the single-attribute functions $v_{n}(n)$ and $v_{d}(d)$ are linear in their arguments. This implies a very specific preference structure, where the value of a change in attribute level from $d^{\prime }$ to $d^{\prime }+\Delta$ is independent of the starting level $d^{\prime }$ ; and likewise for attribute n . Attribute b only has two levels and therefore $v_{b}$ does not require any further specification. For ease of notation we will use the following single-attribute value functions:

$$v_{n}(n)=k_{n}n,\;v_{d}(d)=k_{d}d,\;v_{s}(b)=k_{b}b$$

The scaling parameters $k_{n},k_{d}$ and $k_{b}$ can be elicited as follows. First, we ask for the change in outcome $\hat{d}$ such that we would be indifferent between the current standard treatment and the intervention under study. That is, we require $\hat{d}$ such that

$$\left(n,d=\hat{d},b=0)∼(n,d=0,b=1\right)$$

Secondly, we ask the decision-maker to specify an additional change in outcome $\bar{d}$ that, when added to $\hat{d}$ , would justify increasing the total sample size from 0 to some value $n^{*}$ . That is, we require $\bar{d}$ such that

$$\left(n=0,d=\hat{d},b=0)∼(n=n_{*},d=\hat{d}+\bar{d},b=0\right)$$

Note that the linear nature of $v_{n}$ and $v_{d}$ mean the specific choice of $n_{*}$ is arbitrary. Finally, since value functions are invariant under linear transformations, we can set $k_{n}+k_{d}+k_{b}=1$ and are left with the following system of equations:

$$\begin{array}{rl} k_{d}\hat{d}-k_{b} & =0\\ k_{d}\bar{d}+k_{n}n_{*} & =0\\ k_{d}+k_{n}+k_{b} & =1 \end{array}$$

This gives the following scaling parameters:

$$k_{d}=1/(1+\hat{d}-\bar{d}/n_{*}),\;k_{n}=-k_{d}\bar{d}/n_{*},\;k_{b}=1-k_{d}-k_{n}$$

#### Utility

The value function represents preferences under conditions of certainty, but in reality we are uncertain about the true values of all three attributes. To accommodate this uncertainty, we move from a value function to a utility function. This allows us to compare probability distributions over the attribute space, as opposed to only fixed points, and make decisions by choosing the distribution which has the largest expected utility.

To define the form of our utility function, we first argue that the change in outcome d is *utility independent* of the other two attributes, n and b .

Definition (Utility independence (Keeney and Raiffa,^13^ p. 226)) Attributes Y is utility independent of attribute Z when conditional preferences for lotteries on Y given $z^{\prime }$ do not depend on the particular level of $z^{\prime }$ .

This means that regardless of how much we have sampled, or whether or not we have incurred the treatment costs associated with moving to the intervention, our preferences for gambles on the change in outcome will be the same. Given this assumption together with the additive form of the value function, the utility function must have one of the following forms (Keeney and Raiffa,^13^ p. 330):

$$u(n,d,b)=\left\{ \begin{array}{ll} 1-e^{-\rho v(n,d,b)}, & \rho >0\\ v(n,d,b), & \rho =0\\ -1+e^{-\rho v(n,d,b)}, & \rho <0 \end{array}\right.$$

A value of ρ=0 implies a risk-neutral attitude, whereas ρ greater than (less than) 0 implies a risk-averse (risk-seeking) attitude. To elicit ρ we first note that we can think about gambles on attribute d whilst ignoring the value of the other attributes, since d is utility independent of n and b . We then elicit the value $d^{*}$ such that we would be indifferent between the following:

**Table 4.** A summary of elicited quantities and utility function parameters alongside their interpretations.

Quantity	Question	Parameter	Interpretation
$d^{*}$	What guaranteed d is equal to a 50/50 gamble between (arbitrary) $d_{\min}$ and $d_{\max}$ ?	ρ	Attitude to risk
$\bar{d}$	What d would justify an increase in sample size from 0 to (arbitrary) $n_{*}$ ?	$k_{n}$	Cost of sampling
$\hat{d}$	What d would justify switching from the control to experimental treatments?	$k_{b}$	Cost of experimental treatment
		$k_{d}$	Benefit of outcome improvement

1. Obtaining $d^{*}$ with certainty.
2. A gamble which will result in $d_{\min}$ with probability 0.5 and $d_{\max}$ with probability 0.5.

That is, we find $d^{*}$ such that

$$u(n,d^{*},b)=0.5u(n,d_{\min},b)+0.5u(n,d_{\max},b)$$

In the special case of risk-neutrality, $\rho =0\Leftrightarrow d^{*}=0.5d_{\min}+0.5d_{\max}$ . For ρ≠0 , we have

(8)
$$d^{*}=-\frac{1}{\rho }\ln\;\left(0.5e^{-\rho d_{\min}}+0.5e^{-\rho d_{\max}}\right)$$

and so we can determine ρ given $d^{*}$ . For example, setting (arbitrarily) $d_{\min}=0,d_{\max}=1$ , a value of ρ=2 corresponds with $d^{*}=0.283$ . That is, ρ=2 implies an indifference between obtaining a guaranteed change in outcome of 0.283, and a simple 50/50 gamble between no change at all and a change of 1. Note that the value of ρ obtained in this manner will be specific to the scale used to measure the effect d . A summary of the elicited quantities and utility function parameters is given in Table 4.

Examining the asymptotic behaviour in equation (.1) can help us further understand the proposed utility function. For example, note that as $d_{\max}\to \infty$ ,

$$d^{*}\to d_{\min}-\frac{1}{\rho }\ln\;(0.5)$$

For example, taking $d_{\min}=0$ and ρ=2 as before, $d^{*}$ will tend to 0.347 . This tells us that for a sufficiently large $d_{\max}$ the decision-maker will become (almost) indifferent to the 50/50 gamble between $d_{\min}$ and $d_{\max}$ , and the 50/50 gamble between $d_{\min}$ and $d_{\max}+\Delta$ for some large Δ . This implies that the extra Δ is of no additional value; that once we start reasoning about sufficiently large treatment effects, further improvements have very limited value in comparison to the value of moving away from $d_{\min}$ initially. We can also see that $d^{*}$ will tend to the same limit when $d_{\min}\to -\infty$ , and to $d_{\min}$ ( $d_{\max}$ ) as ρ tends to ∞ ( −∞ ). While considering these asymptotic can help the decision-maker reason about the general behaviour of the utility function, they may nevertheless wish to focus on its behaviour over the plausible region of the attribute space as defined by the joint prior distribution.

### Internal pilot covariance

Recall that we have sample means from both the first and second stages of an internal pilot design, $x_{1},x_{2}$ , with the combined sample mean $x_{t}=n_{1}x_{1}/\left(n_{1}+n_{2}\right)+n_{2}x_{2}/\left(n_{1}+n_{2}\right)$ being used at the final testing stage. The distribution of the combined mean, conditional on μ is $x_{t}|\mu ∼N(\mu ,2\sigma^{2}/\left(n_{1}+n_{2}\right))$ . To fully specify the joint distribution of $\left(x_{1},x_{t}\right)$ , we require the covariance:

$$\mathrm{cov}(x_{1},x_{t})=E[x_{1}x_{t}]-E[x_{1}]E[x_{t}]$$

By the law of total expectation,

$$\begin{array}{rl} E[x_{1}x_{t}] & =E(E[x_{1}x_{t}\;|\;x_{1}])\\ & =E_{(}x_{1}E[x_{t}\;|\;x_{1}])\\ & =E\left(x_{1}E\left[\frac{n_{1}x_{1}}{n_{1}+n_{2}}+\frac{n_{2}x_{2}}{n_{1}+n_{2}}\;|\;x_{1}\right]\right)\\ & =E\left(\frac{n_{1}x_{1}^{2}}{n_{1}+n_{2}}+\frac{n_{2}x_{1}\mu }{n_{1}+n_{2}}\right)\\ & =\frac{n_{1}}{n_{1}+n_{2}}E[x_{1}^{2}]+\frac{n_{2}}{n_{1}+n_{2}}E[x_{1}]\mu \\ & =\frac{n_{1}}{n_{1}+n_{2}}(var(x_{1})+E[x_{1}{]}^{2})+\frac{n_{2}}{n_{1}+n_{2}}\mu^{2}\\ & =\frac{n_{1}}{n_{1}+n_{2}}\left(\frac{2\sigma^{2}}{n_{1}}+\mu^{2}\right)+\frac{n_{2}}{n_{1}+n_{2}}\mu^{2} \end{array}$$

Then,

$$\begin{array}{rl} \mathrm{cov}(x_{1},x_{t}) & =\frac{n_{1}}{n_{1}+n_{2}}\left(\frac{2\sigma^{2}}{n_{1}}+\mu^{2}\right)+\frac{n_{2}}{n_{1}+n_{2}}\mu^{2}-\mu^{2}\\ & =\frac{2\sigma^{2}}{n_{1}+n_{2}}+\frac{n_{1}\mu^{2}}{n_{1}+n_{2}}+\frac{n_{2}\mu^{2}}{n_{1}+n_{2}}-\mu^{2}\\ & =\frac{2\sigma^{2}}{n_{1}+n_{2}} \end{array}$$
