# Supplementary material for: Optimising error rates in programmes of pilot and definitive trials using Bayesian statistical decision theory
Source: Stat Methods Med Res. 2025 Mar 31;34(6):1162–77. doi: 10.1177/09622802251322987 (PMC12209550; doi:10.1177/09622802251322987)
Supplement: sj-pdf-1-smm-10.1177_09622802251322987 - Supplemental material for Optimising error rates in programmes of pilot and definitive trials using Bayesian statistical decision theory [file sj-pdf-1-smm-10.1177_09622802251322987.pdf]

Optimising error rates in programmes of pilot and definitive trials using Bayesian statistical decision theory - supplementary material

D. T. Wilson

18 June, 2024

This document provides the code implementing the methods described in the associated manuscript and reproduces all its figures and results.

Methods

Value

As given in the paper, we use a value function which has three attributes: n (the total sample size), d (the change in mean outcome following the pilot and main trial programme), and b (an indicator that the experimental treatment is not adopted over the control).

$$ v(n, d, s) = k_n n + k_d d + k_b b. $$

To start, we use the elicited preference parameters $\bar{d}, \hat{d}$ and n_* to obtain the weights k_n, k_d and k_b:

```
get_ks <- function(d_bar, d_hat, n)
{
  k_d <- 1/(1 + d_hat - d_bar/n)
  k_n <- -k_d*d_bar/n
  k_b <- 1 - k_d - k_n

  return(c(k_d, k_n, k_b))
}
```

Utility

We use an exponential utility parameterised by \(\rho\), which encodes the decision maker’s attitude to risk.

- \(u(n, d, b) = 1 - e^{-\rho v(n, d, b)}, \rho > 0\),
- \(u(n, d, b) = v(n, d, b), \rho = 0\).
- \(u(n, d, b) = -1 + e^{-\rho v(n, d, b)}, \rho > 0\),

MEU for an external pilot and confirmatory trial programme

To calculate expected utility, we fist find the expected utility conditional on the true treatment effect \(\mu\):

```
# Condition on mu first
```

```
exp_u_mu <- function(mu, n1, d1, n2, d2, k, rho, mu_0, sd_0, sig)
{
  # Calculate power of the pilot and main trial when using critical values
  # d1 and d2 and sample sizes n1 and n2
  pow1 <- 1-pnorm(d1, mu, sqrt(2*sig^2/n1))
  pow2 <- 1-pnorm(d2, mu, sqrt(2*sig^2/n2))

  su <- 0 # set_up

  if(rho > 0){
    pow1*pow2*(1-exp(-rho*(k[1]*mu + k[2]*(n1+n2+su)))) +
    pow1*(1-pow2)*(1-exp(-rho*(k[2]*(n1+n2+su) + k[3]))) +
    (1-pow1)*(1-exp(-rho*(k[2]*n1 + k[3])))
  } else if(rho < 0) {
    pow1*pow2*(-1+exp(-rho*(k[1]*mu + k[2]*(n1+n2+su)))) +
    pow1*(1-pow2)*(-1+exp(-rho*(k[2]*(n1+n2+su) + k[3]))) +
    (1-pow1)*(-1+exp(-rho*(k[2]*n1 + k[3])))
  } else {
    pow1*pow2*(k[1]*mu + k[2]*(n1+n2+su)) +
    pow1*(1-pow2)*(k[2]*(n1+n2+su) + k[3]) +
    (1-pow1)*(k[2]*n1 + k[3])
  }
}

# For example,
d1 <- d2 <- 0.14
n1 <- n2 <- 20
mu_0 <- 0; sd_0 <- 0.6; sig <- 1.5
```

```
rho <- 2;
d_bar <- 0.005; d_hat <- 0.3

k <- get_ks(d_bar, d_hat, n=50)

exp_u_mu(0.2, n1, d1, n2, d2, k, rho, mu_0, sd_0, sig)
```

```
## [1] 0.3347484
```

We are then left with integrating out the \(\mu\):

```
# Set up the quadrature points and weights
rule <- gaussHermiteData(100)
rule$x <- rule$x*sqrt(2)*sd_0 + mu_0

exp_u_joint <- function(x, k, rho, mu_0, sd_0, sig, rule, pen = FALSE)
{
  n1 <- x[1]; d1 <- x[2]; n2 <- x[3]; d2 <- x[4]
  u <- ghQuad(f=exp_u_mu, rule=rule, n1=n1, d1=d1, n2=n2, d2=d2,
        k=k, rho=rho, mu_0=mu_0, sd_0=sd_0, sig=sig)/sqrt(pi)

  # Use an optional penalty to programmes with a pilot larger than the main trial
  return(-u + pen*100*(n2<n1))
}

# For example,
exp_u_joint(c(5, 0.01, 30, 0.12), k=k, rho=rho, mu_0=mu_0, sd_0=sd_0, sig=sig,
rule=rule)
```

```
## [1] -0.3989716
```

To assist with optimisation we can find the derivatives of this expected utility with respect to our design variables \(n_i, d_i\). Since we are integrating numerically, we need to find the derivatives of the conditional expected utility:

\[\frac{d}{dn} Pr[x > d ~|~\mu] = \frac{d}{dn} \left[1 - \Phi \left(\frac{d-\mu}{\sqrt{2\sigma^2/n}} \right)\right] = -\phi\left(\frac{d-\mu}{\sqrt{2\sigma^2/n}} \right) \frac{d-\mu}{2\sqrt{2\sigma^2/n}} \]

\[\frac{d}{dd} Pr[x > d ~|~\mu] = \frac{d}{dd} \left[1 - \Phi \left(\frac{d-\mu}{\sqrt{2\sigma^2/n}} \right)\right] = -\phi\left(\frac{d-\mu}{\sqrt{2\sigma^2/n}} \right) \frac{1}{\sqrt{2\sigma^2/n}} \] Re-writing the expected conditional utility:

\[\begin{aligned} f(n_1,n_2,d_1,d_2) = &g(n_1,d_1)g(n_2,d_2) - g(n_1,d_1)g(n_2,d_2)\exp(-\rho(k_d\mu + k_n (n_1+n_2)) +\\

&g(n_1,d_1)\bar{g}(n_2,d_2) - g(n_1,d_1)\bar{g}(n_2,d_2)\exp(-\rho(k_n (n_1+n_2) + k_c)) + \\ &\bar{g}(n_1,d_1) - \bar{g}(n_1,d_1)\exp(-\rho(k_n n_1 + k_c)) \end{aligned} \]

Implementing this gives:

```
g <- function(n,d,mu,sig)
{
  1 - pnorm((d-mu)/sqrt(2*sig^2/n))
}

g_dn <- function(n,d,mu,sig)
{
  -dnorm((d-mu)/sqrt(2*sig^2/n))*(d-mu)/(2*sqrt(2*n*sig^2))
}

g_dd <- function(n,d,mu,sig)
{
  -dnorm((d-mu)/sqrt(2*sig^2/n))/sqrt(2*sig^2/n)
}

f_dn1 <- function(n1,d1,n2,d2,mu,sig,k,rho)
{
  g_dn(n1,d1,mu,sig)*g(n2,d2,mu,sig) - g(n2,d2,mu,sig)*(g_dn(n1,d1,mu,sig)*exp(-rho*(k[1]*mu + k[2]*(n1+n2))) + g(n1,d1,mu,sig)*exp(-rho*(k[1]*mu + k[2]*(n1+n2)))*(-rho*k[2])) +
    g_dn(n1,d1,mu,sig)*(1-g(n2,d2,mu,sig)) - (1-g(n2,d2,mu,sig))*(g_dn(n1,d1,mu,sig)*exp(-rho*( k[2]*(n1+n2)+k[3])) + g(n1,d1,mu,sig)*exp(-rho*( k[2]*(n1+n2)+k[3]))*(-rho*k[2])) +
    (-g_dn(n1,d1,mu,sig)) - (-g_dn(n1,d1,mu,sig))*exp(-rho*(k[2]*n1+k[3])) - (1-g(n1,d1,mu,sig))*exp(-rho*(k[2]*n1+k[3]))*(-rho*k[2])
}

f_dn2 <- function(n1,d1,n2,d2,mu,sig,k,rho)
{
  g(n1,d1,mu,sig)*g_dn(n2,d2,mu,sig) - g(n1,d1,mu,sig)*(g_dn(n2,d2,mu,sig)*exp(-rho*(k[1]*mu + k[2]*(n1+n2))) + g(n2,d2,mu,sig)*exp(-rho*(k[1]*mu + k[2]*(n1+n2)))*(-rho*k[2])) +
    g(n1,d1,mu,sig)*(-g_dn(n2,d2,mu,sig)) - g(n1,d1,mu,sig)*( (-g_dn(n2,d2,mu,sig))*exp(-rho*( k[2]*(n1+n2)+k[3])) + (1-g(n2,d2,mu,sig))*exp(-rho*( k[2]*(n1+n2)+k[3]))*(-rho*k[2]))
}

f_dd1 <- function(n1,d1,n2,d2,mu,sig,k,rho)
{
```

```
  g_dd(n1,d1,mu,sig)*g(n2,d2,mu,sig) - g_dd(n1,d1,mu,sig)*g(n2,d2,mu,sig)*exp(-rho*(k[1]*mu + k[2]*(n1+n2))) +
    g_dd(n1,d1,mu,sig)*(1-g(n2,d2,mu,sig)) - g_dd(n1,d1,mu,sig)*(1-g(n2,d2,mu,sig))*exp(-rho*( k[2]*(n1+n2)+k[3])) +
    (- g_dd(n1,d1,mu,sig)) - (- g_dd(n1,d1,mu,sig))*exp(-rho*(k[2]*n1+k[3]))
}

f_dd2 <- function(n1,d1,n2,d2,mu,sig,k,rho)
{
  g(n1,d1,mu,sig)*g_dd(n2,d2,mu,sig) - g(n1,d1,mu,sig)*g_dd(n2,d2,mu,sig)*exp(-rho*(k[1]*mu + k[2]*(n1+n2))) +
    g(n1,d1,mu,sig)*(-g_dd(n2,d2,mu,sig)) - g(n1,d1,mu,sig)*(-g_dd(n2,d2,mu,sig))*exp(-rho*( k[2]*(n1+n2)+k[3]))
}
```

We can check this against numerical methods:

```
exp_u_joint_grad <- function(x, k, rho, mu_0, sd_0, sig, rule, pen=FALSE)
{
  n1 <- x[1]; d1 <- x[2]; n2 <- x[3]; d2 <- x[4]
  g_n1 <- ghQuad(f=f_dn1, rule=rule, n1=n1, d1=d1, n2=n2, d2=d2,k=k, rho=rho, sig=sig)/sqrt(pi)
  g_d1 <- ghQuad(f=f_dd1, rule=rule, n1=n1, d1=d1, n2=n2, d2=d2,k=k, rho=rho, sig=sig)/sqrt(pi)
  g_n2 <- ghQuad(f=f_dn2, rule=rule, n1=n1, d1=d1, n2=n2, d2=d2,k=k, rho=rho, sig=sig)/sqrt(pi)
  g_d2 <- ghQuad(f=f_dd2, rule=rule, n1=n1, d1=d1, n2=n2, d2=d2,k=k, rho=rho, sig=sig)/sqrt(pi)
  return(c(g_n1, g_d1, g_n2, g_d2)*(-1*(rho > 0) + 1*(rho < 0)))
}

n1 <- 10; d1 <- 0.1; n2 <- 15; d2 <- 0
mu <- 0.1; rho <- -1

grad(exp_u_joint, c(n1,d1,n2,d2), k=k, rho=rho, mu_0=mu_0, sd_0=sd_0, sig=sig, rule=rule)
```

```
## [1] -0.0016531690  0.0032361184 -0.0007830335 -0.0133222608
```

```
exp_u_joint_grad(c(n1,d1,n2,d2), k=k, rho=rho, mu_0=mu_0, sd_0=sd_0, sig=sig, rule=rule)
```

```
## [1] -0.0016531690  0.0032361183 -0.0007830335 -0.0133222608
```

Illustration

Using the values of the OK-Diabetes example, we can find our optimal programme. We do this in the unrestricted case first, and then when we force the pilot trial to have a type I error rate of 1.

```
mu_0 <- 0; sd_0 <- 0.6; sig <- 1.5; mu_1 <- 0.5
```

```
rho <- 2;
d_bar <- 0.005; d_hat <- 0.3

k <- get_ks(d_bar, d_hat, n=50)
rule <- gaussHermiteData(100)
rule$x <- rule$x*sqrt(2)*sd_0 + mu_0

# Choose a starting point for the optimisation
x <- c(100, 0, 100, 0)

opt <- optim(x, exp_u_joint, gr = exp_u_joint_grad,
             lower = c(30,-40,0,-40), upper = c(1000,40,1000,40), method = "L-BFGS-
B",
             k=k, rho=rho, mu_0=mu_0, sd_0=sd_0, sig=sig, rule=rule, pen=T)

x <- opt$par

r1 <- c(x,
         1 - pnorm(x[2]/sqrt(2*(sig^2)/x[1])),
         pnorm(x[2], mu_1, sqrt(2*sig^2/x[1])),
         1 - pnorm(x[4]/sqrt(2*(sig^2)/x[3])),
         pnorm(x[4], mu_1, sqrt(2*sig^2/x[3])),
        opt$value)

# Force a type I of 1 by constraining to low d_1
x <- c(100, -39, 100, 0)

opt <- optim(x, exp_u_joint, gr = exp_u_joint_grad,
             lower = c(30,-40,0,-40), upper = c(1000,-39,1000,40), method = "L-BFGS-
B",
             k=k, rho=rho, mu_0=mu_0, sd_0=sd_0, sig=sig, rule=rule, pen=F)

x <- opt$par

r2 <- c(x,
         1 - pnorm(x[2]/sqrt(2*(sig^2)/x[1])),
         pnorm(x[2], mu_1, sqrt(2*sig^2/x[1])),
         1 - pnorm(x[4]/sqrt(2*(sig^2)/x[3])),
```

```
      pnorm(x[4], mu_1, sqrt(2*sig^2/x[3])),
     opt$value)
r2
```

```
## [1]  30.00000000 -39.00000000 109.62027938   0.36578868
1.00000000
## [6]   0.00000000   0.03550735   0.25385353  -0.42292113
```

```
# Tabulate and present the results
tab <- as.data.frame(rbind(r1, r2))

tab <- data.frame(pr = c("Unrestricted", "No pilot test"),
                  n_1=round(tab[,1]), n_2=round(tab[,3]),
                  a_1 = round(tab[,5], 2), p_1 = round(tab[,6], 3),
                  a_2 = round(tab[,7], 3), p_2 = round(tab[,8], 3),
                  u = round(tab[,9], 5)
                  )
colnames(tab) <- c("Problem", "$n_1$", "$n_2$", "$\\alpha_1$", "$\\beta_1$",
"$\\alpha_2$", "$\\beta_2$", "Expected utility")
#tab[2,2] <- 0
tab
```

```
##          Problem $n_1$ $n_2$ $\\alpha_1$ $\\beta_1$ $\\alpha_2$
$\\beta_2$
## 1  Unrestricted    41   146        0.39       0.11       0.041
0.132
## 2 No pilot test    30   110        1.00       0.00       0.036
0.254
##   Expected utility
## 1         -0.42874
## 2         -0.42292
```

```
#print(xtable(tab, digits = c(1,0,0,0,2,3,3,3,5)), booktabs = T,
include.rownames = F,
#      sanitize.text.function = function(x) {x}, floating = F,
#      file = "./paper/tables/ill.txt")
```

Translate the difference in utility back into the basic units of treatment difference and sample size:

```
u1 <- 0.42292
u2 <- 0.42874
v1 <- log(1-u1)/(-rho); v2 <- log(1-u2)/(-rho)
```

```
v_dif <- v1-v2
v_dif/k
```

```
## [1] -0.006588191 65.881905859 -0.021960635
```

Sensitivity analysis

For the optimal design for the full programme as found above, how robust is it to deviations in the parameters of our utility and our prior?

```
mu_0 <- 0; sd_0 <- 0.6; sig <- 1.5; mu_1 <- 0.5
```

```
rho <- 2; d_bar <- 0.005; d_hat <- 0.3

k <- get_ks(d_bar, d_hat, n=50)
rule <- gaussHermiteData(100)
rule$x <- rule$x*sqrt(2)*sd_0 + mu_0

des <- r1[1:4]

sens_f <- function(pars, des)
{
  mu_0 <- pars[1]; sd_0 <- pars[2]; sig <- pars[3]
  rho <- pars[4]; d_bar <- pars[5]; d_hat <- pars[6]

  k <- get_ks(d_bar, d_hat, n=50)
  rule <- gaussHermiteData(100)
  rule$x <- rule$x*sqrt(2)*sd_0 + mu_0

  old_u <- -exp_u_joint(x=des, k=k, rho=rho, mu_0=mu_0, sd_0=sd_0, sig=sig,
rule=rule, pen=F)

  x <- c(100, 0, 100, 0)

  opt <- optim(x, exp_u_joint, gr = exp_u_joint_grad,
            lower = c(30,-40,0,-40), upper = c(1000,40,1000,40), method = "L-BFGS-
B",
            k=k, rho=rho, mu_0=mu_0, sd_0=sd_0, sig=sig, rule=rule, pen=F)

  x <- opt$par

  new_u <- -opt$value

  opt <- nloptr(x, exp_u_joint,
               lb = c(30,-40,0,-40), ub= c(1000,40,1000,40),
```

```
              opt = list("algorithm"="NLOPT_LN_SBPLX",
                         "xtol_rel"=1.0e-8,
                         "maxeval"=5000),
              k=k, rho=rho, mu_0=mu_0, sd_0=sd_0, sig=sig, rule=rule, pen=F)

  new_u <- -opt$objective

  equiv_n <- (log(1-new_u)/(-rho) - log(1-old_u)/(-rho))/k[2]

  c(old_u, new_u, equiv_n)
}

lims <- data.frame(mu_0 = mu_0 + c(-0.25, 0.25),
               sd_0 = sd_0 + c(-0.12, 0.12),
               sig = sig + c(-0.2, 0.2),
               rho = rho + c(-2, 2),
               d_bar = d_bar + c(-0.004, 0.005),
               d_hat = d_hat + c(-0.2, 0.2)
               )

# Sensitivity to prior params

df_p <- sobol(500, 2)
for(i in 1:2){
  df_p[,i] <- df_p[,i]*(lims[2,i] - lims[1,i]) + lims[1,i]
}
df_p <- as.data.frame(df_p)
df_p$sig <- sig; df_p$rho <- rho; df_p$d_bar <- d_bar; df_p$d_hat <- d_hat
names(df_p) <- names(lims)

df_p <- cbind(df_p, t(apply(df_p, 1, sens_f, des=des)))
names(df_p)[9] <- "dif"

# Sensitivity to utility params
df_u <- as.data.frame(sobol(500, 2))
df_u$mu_0 <- mu_0; df_u$sd_0 <- sd_0; df_u$sig <- sig
df_u <- df_u[,c(3,4,5,1,2)]
for(i in 4:5){
  df_u[,i] <- df_u[,i]*(lims[2,i] - lims[1,i]) + lims[1,i]
}
df_u$d_hat <- d_hat
names(df_u) <- names(lims)

df_u <- cbind(df_u, t(apply(df_u, 1, sens_f, des=des)))
names(df_u)[9] <- "dif"
```

Plot the results:

```
fit_p <- gam(dif ~ te(mu_0, sd_0, k=16), data=df_p) # s(mu_0) +
s(sd_0) +
```

```
to_plot_p <- expand.grid(mu_0 = seq(min(df_p$mu_0), max(df_p$mu_0), l=50),
                         sd_0 = seq(min(df_p$sd_0), max(df_p$sd_0), l=50))
to_plot_p$dif <- -predict(fit_p, newdata = to_plot_p)

ggplot(to_plot_p, aes(mu_0, sd_0, z=dif)) +
  geom_contour_filled(breaks = c(0, 2, 4, 6, 8, 10), alpha = 0.5) +
  scale_fill_brewer(name="Regret", palette = "YlGnBu") +
  theme_minimal() +
  xlab( expression(paste("Prior mean, ", m))) +
  ylab( expression(paste("Prior standard deviation, ", s)))
```

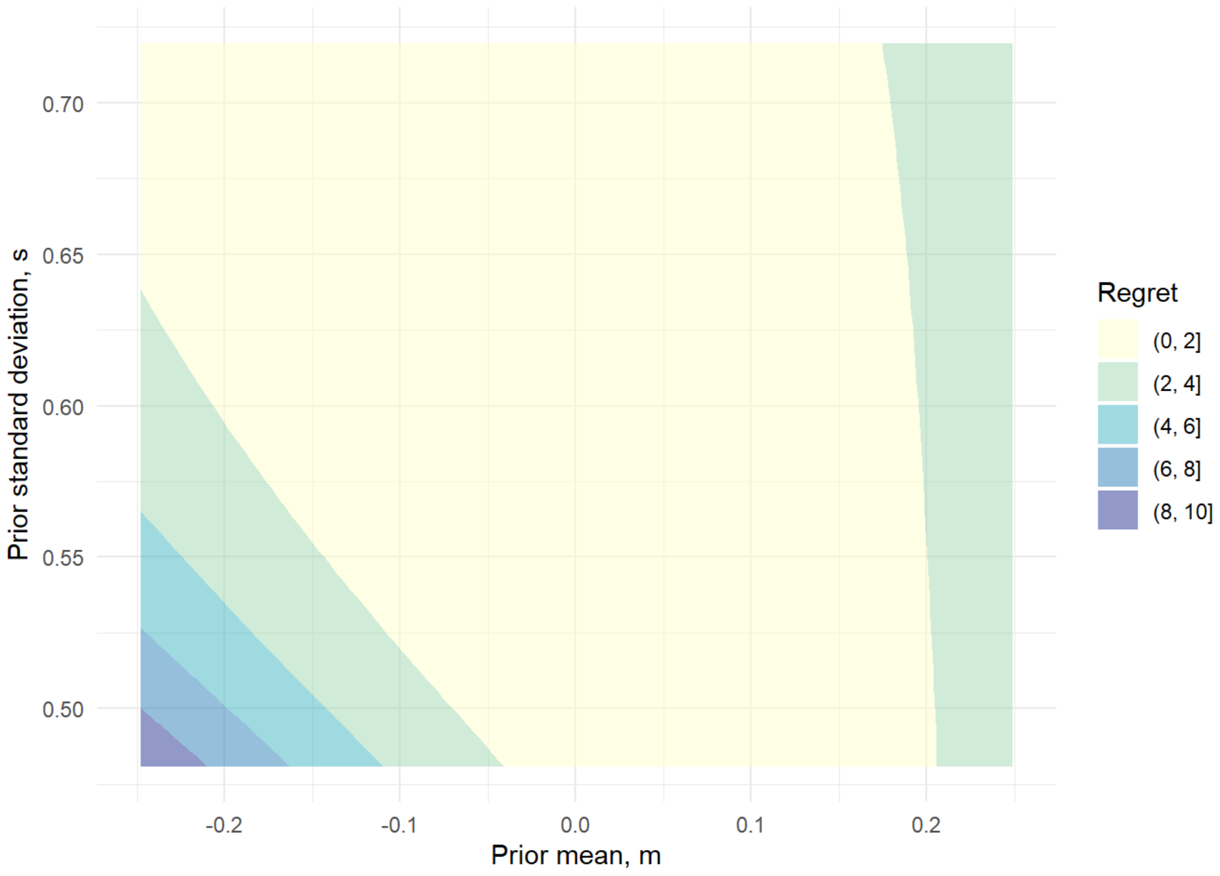


```
#ggsave("./figures/sens_p.pdf", height=9, width=14, units="cm")
```

```
#ggsave("./figures/sens_p.eps", height=9, width=14, units="cm", device =
cairo_ps())
```

```
fit_u <- gam(dif ~ te(rho, d_bar, k=16), data=df_u) # s(mu_0) + s(sd_0) +

to_plot_u <- expand.grid(rho = seq(0,4,l=50),
                         d_bar = seq(0.001,0.01, l=50))
to_plot_u$dif <- -predict(fit_u, newdata = to_plot_u)

ggplot(to_plot_u, aes(rho, d_bar, z=dif)) +
  geom_contour_filled(breaks = seq(0, 45, 5), alpha = 0.5) +
  scale_fill_brewer(name="Regret", palette = "YlGnBu") +
  theme_minimal() +
  xlab( expression(paste("Risk attitude, ", rho))) +
  ylab( expression(paste("Cost of sampling, ", bar(d))))
```

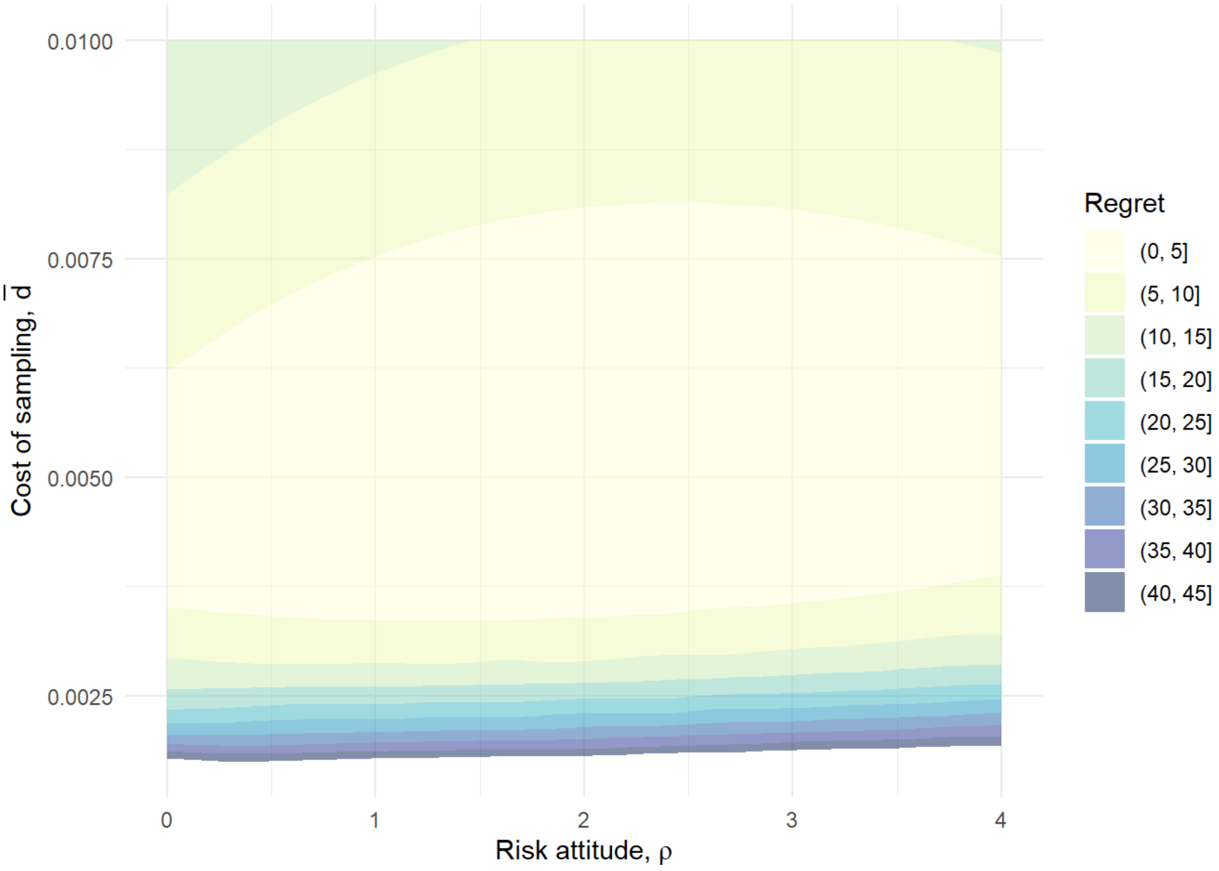


```
#ggsave("./figures/sens_u.pdf", height=9, width=14, units="cm")
```

```
#ggsave("./figures/sens_u.eps", height=9, width=14, units="cm", device =
cairo_ps())
```

Evaluation

We now find optimal designs for a range of problems. We consider two cases. Firstly, we set a lower limit on the pilot sample size of 30, corresponding to cases where we want this sample at least for feasibility questions. Secondly, we remove this lower limit and allow the pilot size to be as small as is optimal, including not doing the pilot at all.

```
mu_0 <- 0; sd_0 <- 0.6; sig <- 1.5; mu_1 <- 0.5
```

```
df <- expand.grid(rho = seq(5, -5, length.out = 100),
                  d_bar = c(0.01, 0.005, 0.0025),
                  d_hat = c(0.1, 0.2, 0.3))



rs <- NULL
x <- c(50, 0.001, 100, 0.001)
for(i in 1:nrow(df)){

  k <- get_ks(df$d_bar[i], df$d_hat[i], n=50)
  rule <- gaussHermiteData(100)
  rule$x <- rule$x*sqrt(2)*sd_0 + mu_0

  if(df$rho[i] == 5) x <- c(50, 0.001, 100, 0.001)
  opt <- optim(x, exp_u_joint, gr = exp_u_joint_grad,
              lower = c(30,-40,0.0001,-40), upper = c(1000,40,1000,40), method = "L-
BFGS-B",
              control = list(factr = 1e5),
              k=k, rho=df$rho[i], mu_0=mu_0, sd_0=sd_0, sig=sig, rule=rule, pen=F)

  x <- opt$par

  r <- c(x, 1-pnorm(x[2]/sqrt(2*(sig^2)/x[1])),
    1-pnorm(x[2], mu_1, sqrt(2*sig^2/x[1])),
    1-pnorm(x[4]/sqrt(2*(sig^2)/x[3])),
    1-pnorm(x[4], mu_1, sqrt(2*sig^2/x[3])),
    opt$value)

  rs <- cbind(rs,r)
  #print(c(df$d_bar[i], df$rho[i]))
  #print(c(r[5:8], opt$value))
  #print(x)
}

df <- cbind(df, t(rs))

names(df) <- c("rho", "d_bar", "d_hat", "n1", "d1", "n2", "d2", "a1", "b1", "a2", "b2",
"u")
```

```
# With no lower bound on pilot n
#saveRDS(df, "../data/opt_joint_unrest.Rda")

# With np at least 30
#saveRDS(df, "../data/opt_joint_np30.Rda")
```

```
#df <- readRDS("../data/opt_joint_unrest.Rda")
```

```
#df <- readRDS("./data/opt_joint_np30.Rda")

df2 <- df
df2$n1 <- df2$n1/500; df2$n2 <- df2$n2/500;
df2 <- melt(df2, id.vars = c("rho", "d_bar", "d_hat"))
df2 <- cbind(df2[substr(df2$variable,2,2) == "1",], df2[substr(df2$variable,2,2) ==
"2",4:5])
names(df2)[4:7] <- c("t1", "v1", "t2", "v2")
df2 <- melt(df2, id.vars = c("rho", "d_bar", "d_hat", "t1", "t2"))
df2 <- df2[,c(1,2,3,4,6,7)]
df2$t1 <- substr(df2$t1,1,1)
df2$variable <- substr(df2$variable,2,2)
names(df2) <- c("rho", "d_bar", "d_hat", "var", "trial", "val")

ggplot(df2[df2$var %in% c("a", "b", "n"),], aes(rho, val, colour=trial, linetype=var))
+
  geom_line() +
  theme_minimal() +
  scale_colour_manual(values=cols) +
  scale_y_continuous(breaks=seq(0,1,0.2)) +
  facet_wrap(d_bar ~ d_hat) + #, labeller = "label_both") + #label_bquote(alpha ^
.(vs))
  xlab(expression(paste("Attitude to risk, ", rho))) +
  ylab("Value") +
  labs(colour = "Stage", linetype = "Variable") +
  theme(panel.spacing = unit(2, "lines"), legend.position="bottom") +
  scale_linetype_manual(values=c(1,2,3), labels=c(expression(alpha), expression(1-
beta), "n/500"))
```

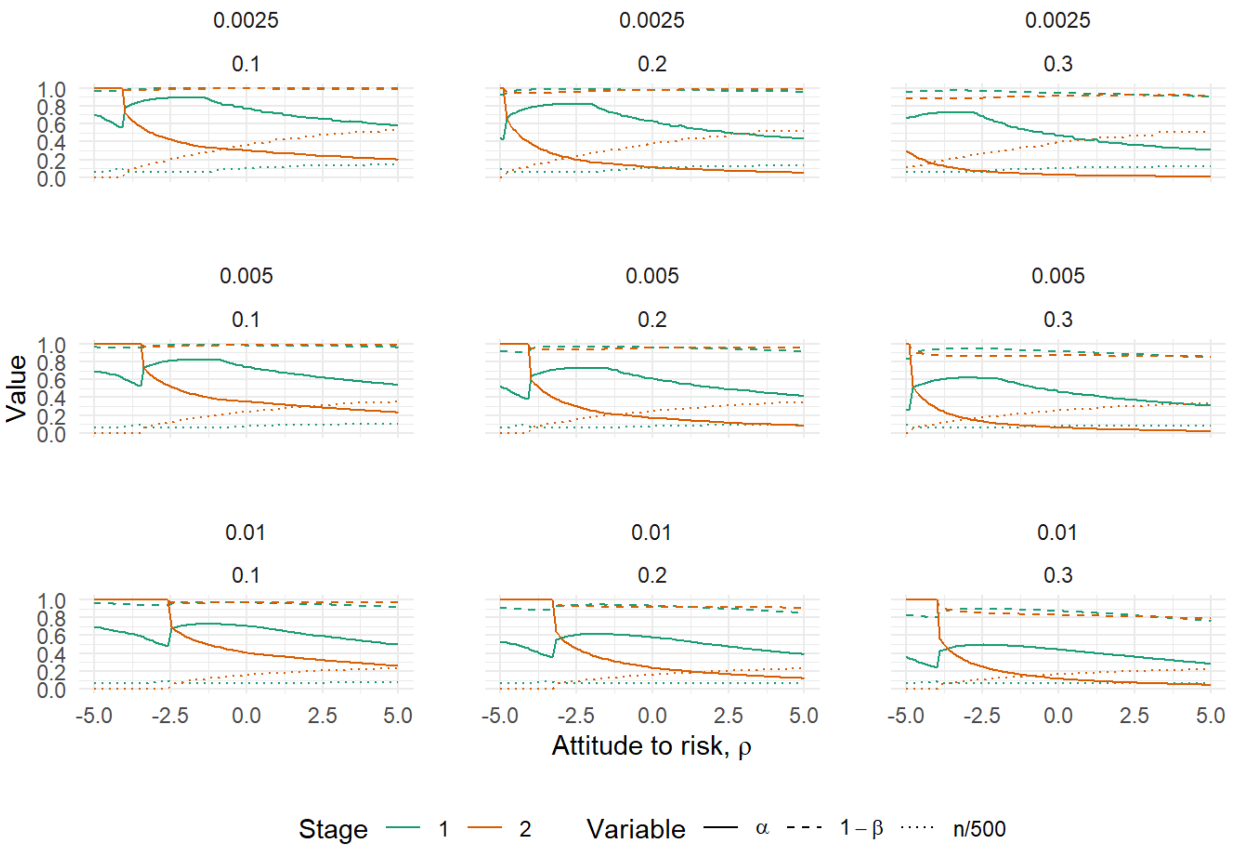


```
#ggsave("./figures/eval_unrest.pdf", height=16, width=18,
units="cm")
```

```
#ggsave("./figures/eval_unrest.eps", height=16, width=18, units="cm", device =
cairo_ps())

#ggsave("./figures/eval_np30.pdf", height=16, width=18, units="cm")
#ggsave("./figures/eval_np30.eps", height=16, width=18, units="cm", device =
cairo_ps())
```

Extensions

Internal pilots

If we have an internal pilot then we will have correlated sample mean statistics at stages 1 and 2. To allow for this we just need to modify the probability terms in the conditional expected utility.

```
# Condition on mu first
```

```
exp_u_mu_int <- function(mu, n1, d1, n2, d2, k, rho, mu_0, sd_0, sig)
{
  cov_m <- matrix(c(2*sig^2/n1, rep(2*sig^2/(n1+n2), 3)), ncol=2)

  gg <- pmvnorm(lower=c(d1, d2), upper=c(Inf, Inf), mean=rep(mu, 2),
sigma=cov_m)[1]
  gs <- pmvnorm(lower=c(d1, -Inf), upper=c(Inf, d2), mean=rep(mu, 2),
sigma=cov_m)[1]
  s <- pmvnorm(lower=c(-Inf, -Inf), upper=c(d1, Inf), mean=rep(mu, 2),
sigma=cov_m)[1]

  if(rho > 0){
    gg*(1-exp(-rho*(k[1]*mu + k[2]*(n1+n2)))) +
    gs*(1-exp(-rho*(k[2]*(n1+n2) + k[3]))) +
    s*(1-exp(-rho*(k[2]*n1 + k[3])))
  } else if(rho < 0) {
    gg*(-1+exp(-rho*(k[1]*mu + k[2]*(n1+n2)))) +
    gs*(-1+exp(-rho*(k[2]*(n1+n2) + k[3]))) +
    s*(-1+exp(-rho*(k[2]*n1 + k[3])))
  } else {
    gg*(k[1]*mu + k[2]*(n1+n2)) +
    gs*(k[2]*(n1+n2) + k[3]) +
    s*(k[2]*n1 + k[3])
  }
}

# For example,
d1 <- d2 <- 0.14
n1 <- n2 <- 20
mu_0 <- 0; sd_0 <- 0.6; sig <- 1.5
rho <- 2;
d_bar <- 0.005; d_hat <- 0.3

k <- get_ks(d_bar, d_hat, n=50)

exp_u_mu_int(0.2, n1, d1, n2, d2, k, rho, mu_0, sd_0, sig)
```

```
## [1] 0.3206075
```

We are then left with integrating out the \(\mu\):

```
exp_u_joint_int <- function(x, k, rho, mu_0, sd_0, sig, rule, pen
= FALSE)
```

```
{
  n1 <- x[1]; d1 <- x[2]; n2 <- x[3]; d2 <- x[4]
```

```
  u <- ghQuad(f=Vectorize(exp_u_mu_int, "mu"), rule=rule, n1=n1, d1=d1, n2=n2,
d2=d2,
         k=k, rho=rho, mu_0=mu_0, sd_0=sd_0, sig=sig)/sqrt(pi)
  # return expected utility and a penalty to avoid having a larger sample in the
  # pilot than in the confirmatory trial
  return(-u + pen*100*(n2<n1))
}

# For example,
exp_u_joint_int(c(5, 0.01, 30, 0.12), k=k, rho=rho, mu_0=mu_0, sd_0=sd_0,
sig=sig, rule=rule)
```

```
## [1] -0.3909082
```

Now we apply this to the illustrative example. Note that we haven't worked out the derivatives for this case, so we take a different approach to optimisation and run first a gradient-free global optimiser and then use its solution as the starting point for a gradient-free local optimiser.

```
mu_0 <- 0; sd_0 <- 0.6; sig <- 1.5; mu_1 <- 0.5
```

```
rho <- 2;
d_bar <- 0.005; d_hat <- 0.3

k <- get_ks(d_bar, d_hat, n=50)
rule <- gaussHermiteData(100)
rule$x <- rule$x*sqrt(2)*sd_0 + mu_0

# Choose a starting point for the optimisation
x <- c(100, 0, 100, 0)
opt <- nloptr(x, exp_u_joint_int,
              lb = c(30,-40,0,-40), ub= c(1000,40,1000,40),
              opt = list("algorithm"="NLOPT_GN_DIRECT",  #"NLOPT_GD_STOGO",
                         "xtol_rel"=1.0e-7,
                         "maxeval"=1000),
              k=k, rho=rho, mu_0=mu_0, sd_0=sd_0, sig=sig, rule=rule, pen=F)

  x <- opt$solution

  opt <- nloptr(x, exp_u_joint_int,
              lb = c(30,-40,0,-40), ub= c(1000,40,1000,40),
              opt = list("algorithm"="NLOPT_LN_SBPLX",
                         "xtol_rel"=1.0e-8,
                         "maxeval"=5000),
              k=k, rho=rho, mu_0=mu_0, sd_0=sd_0, sig=sig, rule=rule, pen=F)
```

```
x <- opt$solution

cov_m <- matrix(c(2*sig*sig/x[1], rep(2*sig*sig/(x[1]+x[3]), 3)), ncol=2)

r2_int <- c(x,
  # prob of passing stage one under null
  pmvnorm(lower=c(x[2], -Inf), upper=c(Inf, Inf), mean=rep(0,2), sigma=cov_m)[1],
  # prob of not passing stage one under alt
  pmvnorm(lower=c(-Inf, -Inf), upper=c(x[2], Inf), mean=rep(0.5,2),
sigma=cov_m)[1],
  # prob of passing both stages under null
  pmvnorm(lower=c(x[2], x[4]), upper=c(Inf, Inf), mean=rep(0,2),
sigma=cov_m)[1],
  # prob of not passing either stage under alt
  pmvnorm(lower=c(-Inf, -Inf), upper=c(x[2], Inf), mean=rep(0.5,2),
sigma=cov_m)[1] + pmvnorm(lower=c(-Inf, -Inf), upper=c(Inf, x[4]),
mean=rep(0.5,2), sigma=cov_m)[1] - pmvnorm(lower=c(-Inf, -Inf), upper=c(x[2],
x[4]), mean=rep(0.5,2), sigma=cov_m)[1],
  opt$objective)

# For comparison, get the overall type I and II error rates for the external design
r1_int <- r1
r1_int[7] <- r1_int[7]*r1_int[5]
r1_int[8] <- r1_int[8] + r1_int[6] - r1_int[8]*r1_int[6]

tab <- as.data.frame(rbind(r1_int, r2_int))

tab <- data.frame(pr = c("External", "Internal"),
                  n_1=round(tab[,1]), n_2=round(tab[,3]),
                  a_1 = round(tab[,5], 2), p_1 = round(tab[,6], 3),
                  a_2 = round(tab[,7], 3), p_2 = round(tab[,8], 3),
                  u = round(tab[,9], 5)
                   )
colnames(tab) <- c("Problem", "$n_1$", "$n_2$", "$\\alpha_1$", "$\\beta_1$",
"$\\alpha_t$", "$\\beta_t$", "Expected utility")
tab
```

```
##    Problem $n_1$ $n_2$ $\\alpha_1$ $\\beta_1$ $\\alpha_t$
$\\beta_t$
## 1 External    41   146        0.39      0.110       0.016
0.228
## 2 Internal    45   121        0.42      0.084       0.016
0.213
##   Expected utility
## 1         -0.42874
```

```
## 2         -0.42954
```

```
#print(xtable(tab, digits = c(1,0,0,0,2,3,3,3,5)), booktabs = T, include.rownames = F,
#      sanitize.text.function = function(x) {x}, floating = F,
#      file = "./tables/ill_int.txt")
```

Heteregeneous effects

We need to change is the calculation of power at the pilot stage in our conditional expected utility, now needing to use the distribution of the pilot estimate conditional on the true main trial effect \(\mu\). For our illustration, we are assuming the pilot and main trial effects have the same marginal priors with means \(m\) and standard deviations \(s\) and a correlation of \(\tau\), giving

\[x_1 ~|~ \mu \sim N\left(m + \tau(\mu - m), (1 - \tau^2)s^2 + \frac{2\sigma^2}{n_1}\right). \]

```
exp_u_mu_bias <- function(mu, n1, d1, n2, d2, k, rho, mu_0, sd_0, sig, cor_pm)
{
  # set variance of main equal to pilot
  var_m <- sd_0^2

  pow1 <- 1 - pnorm(d1, mu_0 + cor_pm*(mu - mu_0)*sqrt(sd_0^2)/sqrt(var_m), sqrt((1 - cor_pm^2)*(sd_0^2) + 2*sig^2/n1))

  pow2 <- 1 - pnorm(d2, mu, sqrt(2*sig^2/n2))

  su <- 0 # set_up

  if(rho > 0){
    pow1*pow2*(1-exp(-rho*(k[1]*mu + k[2]*(n1+n2+su)))) +
    pow1*(1-pow2)*(1-exp(-rho*(k[2]*(n1+n2+su) + k[3]))) +
    (1-pow1)*(1-exp(-rho*(k[2]*n1 + k[3])))
  } else if(rho < 0) {
    pow1*pow2*(-1+exp(-rho*(k[1]*mu + k[2]*(n1+n2+su)))) +
    pow1*(1-pow2)*(-1+exp(-rho*(k[2]*(n1+n2+su) + k[3]))) +
    (1-pow1)*(-1+exp(-rho*(k[2]*n1 + k[3])))
  } else {
    pow1*pow2*(k[1]*mu + k[2]*(n1+n2+su)) +
    pow1*(1-pow2)*(k[2]*(n1+n2+su) + k[3]) +
    (1-pow1)*(k[2]*n1 + k[3])
  }
```

```
}

exp_u_joint_bias <- function(x, k, rho, mu_0, sd_0, sig, cor_pm, rule, pen = FALSE)
{
  n1 <- x[1]; d1 <- x[2]; n2 <- x[3]; d2 <- x[4]
  u <- ghQuad(f=exp_u_mu_bias, rule=rule, n1=n1, d1=d1, n2=n2, d2=d2,
         k=k, rho=rho, mu_0=mu_0, sd_0=sd_0, sig=sig,
         cor_pm=cor_pm)/sqrt(pi)

  return(-u + pen*100*(n2<n1))
}

mu_0 <- 0; sd_0 <- 0.6; sig <- 1.5; mu_1 <- 0.5;

# Use utility parameter values from the illustrative example
rho <- -2; d_bar <- 0.005; d_hat <- 0.3

# Find optimal designs for different correlations
df <- data.frame(cor_pm = seq(0.6, 1, 0.01))

rs <- NULL
x <- c(50, 0.001, 100, 0.001)
for(i in 1:nrow(df)){

  k <- get_ks(d_bar, d_hat, n=50)
  rule <- gaussHermiteData(100)
  rule$x <- rule$x*sqrt(2)*sqrt(sd_0^2) + mu_0

  opt <- optim(x, exp_u_joint_bias, #gr = exp_u_joint_grad,
             lower = c(30,-41,0.0001,-40), upper = c(1000, 40,1000,40), method = "L-
BFGS-B",
             control = list(factr = 1e5),
             k=k, rho=rho, mu_0=mu_0, sd_0=sd_0, sig=sig, cor_pm=df$cor_pm[i],
             rule=rule, pen=F)

  x <- opt$par

  r <- c(x, 1-pnorm(x[2]/sqrt(2*(sig^2)/x[1])),
    1-pnorm(x[2], mu_1, sqrt(2*sig^2/x[1])),
    1-pnorm(x[4]/sqrt(2*(sig^2)/x[3])),
    1-pnorm(x[4], mu_1, sqrt(2*sig^2/x[3])),
    opt$value)

  rs <- cbind(rs,r)
}
```

```
df <- cbind(df, t(rs))

names(df) <- c("cor_pm", "n1", "d1", "n2", "d2", "a1", "b1", "a2", "b2", "u")
```

Output the results for the illustrative example where \(\tau = 0.9\) and the previous case of perfect correlation for comparison:

```
tab <- df[c(31, 41), ]
```

```
tab <- data.frame(cor_pm = tab[,1],
                  n_1=round(tab[,2]), n_2=round(tab[,4]),
                  a_1 = round(tab[,6], 2), p_1 = round(tab[,7], 3),
                  a_2 = round(tab[,8], 3), p_2 = round(tab[,9], 3),
                  u = round(tab[,10], 5)
                  )

colnames(tab) <- c("$\\tau$", "$n_1$", "$n_2$", "$\\alpha_1$", "$\\beta_1$",
"$\\alpha_2$", "$\\beta_2$", "Expected utility")
tab
```

```
##   $\\tau$ $n_1$ $n_2$ $\\alpha_1$ $\\beta_1$ $\\alpha_2$
$\\beta_2$
```

```
## 1     0.9    30    97         0.84      0.989        0.091
0.839
## 2     1.0    30    97         0.61      0.942        0.108
0.861
##   Expected utility
## 1         -1.04861
## 2         -1.05344
```

```
#print(xtable(tab, digits = c(1, 1,0,0,2,3,3,3,5)), booktabs = T,
include.rownames #= F,
```

```
#      sanitize.text.function = function(x) {x}, floating = F,
#      file = "./tables/corr.txt")
```

Plot the optimal designs over the range of \(\tau\):

```
df2 <- df
```

```
df2$n1 <- df2$n1/500; df2$n2 <- df2$n2/500;
df2 <- df2[,-c(3, 5, 10)]

df2 <- melt(df2, id.vars = c("cor_pm"))
df2 <- cbind(df2, substr(df2$variable,2,2), substr(df2$variable,1,1))
names(df2)[4:5] <- c("t", "v")
```

```
ggplot(df2, aes(cor_pm, value, colour=t, linetype=v)) +
  geom_line() +
  theme_minimal() +
  scale_colour_manual(values=cols) +
  xlab(expression(paste("Effect correlation, ", tau))) +
  ylab("Value") +
  labs(colour = "Stage", linetype = "Variable") +
  scale_linetype_manual(values=c(1,2,3), labels=c(expression(alpha), expression(1-
beta), "n/500"))
```

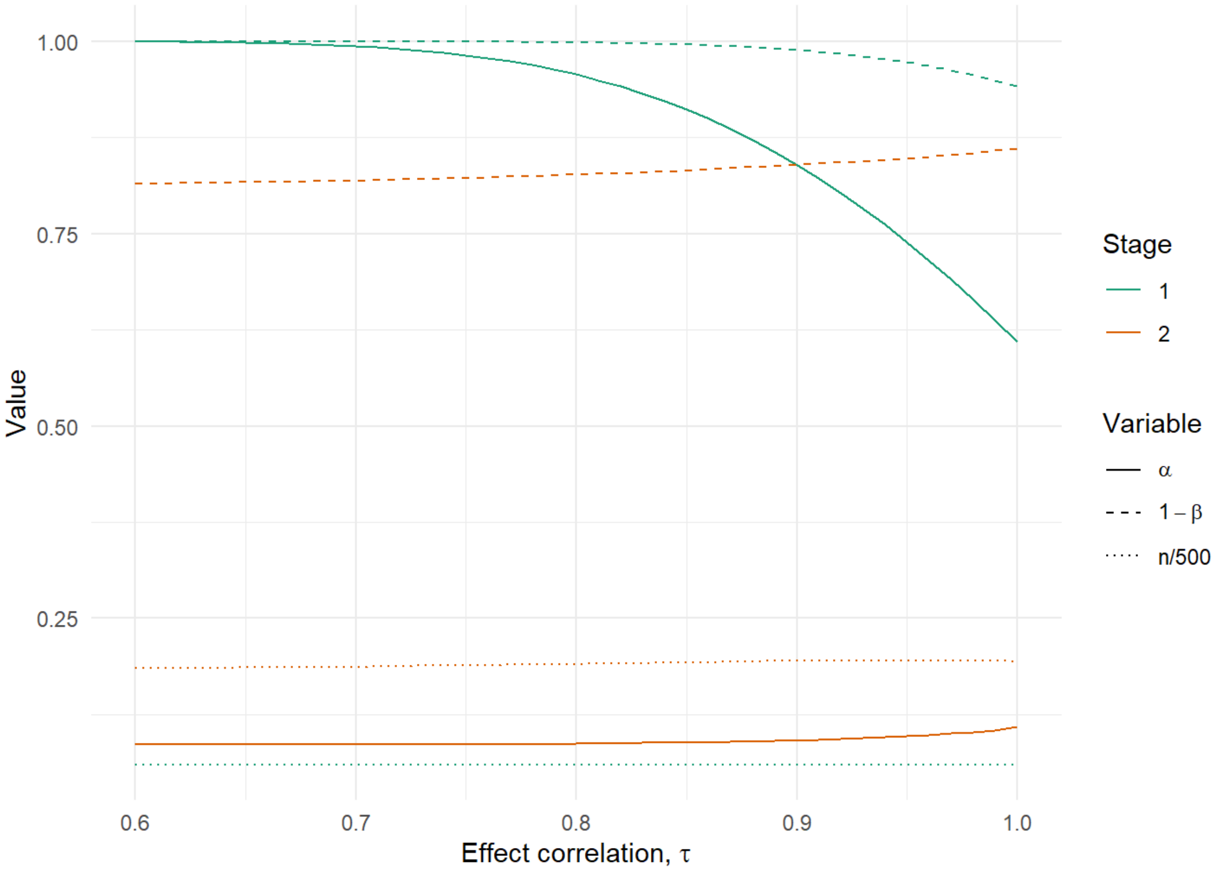


```
#ggsave("./figures/corr.pdf", height=9, width=14, units="cm")
```

```
#ggsave("./figures/corr.eps", height=9, width=14, units="cm", device = cairo_ps())
```

Note that the utility of the programme will range between the unrestrictied and no-pilot-testing examples earlier, since the former assumed perfect correlation and the latter strategy becomes optimal when the correlation is weak.

Figures

```
df <- expand.grid(a = seq(0,1,0.001),
                  n = c(35))

df$b <- apply(df, 1, function(x) 1 - power.t.test(n=x[2], delta=0.5, sd=1.5, sig.level
= x[1], alternative = "o")$power)

ggplot(df, aes(a,b)) + geom_line() +
  coord_fixed() +
  xlab(expression(paste("Type I error rate, ", alpha))) +
  ylab(expression(paste("Type II error rate, ", beta))) +
  theme_minimal() +
  geom_point(data=df[df$a == 0.75 & df$n == 35,], colour=cols[3], size=2) +
  geom_point(data=df[df$a == 1 & df$n == 35,], colour=cols[2], size=2)
```

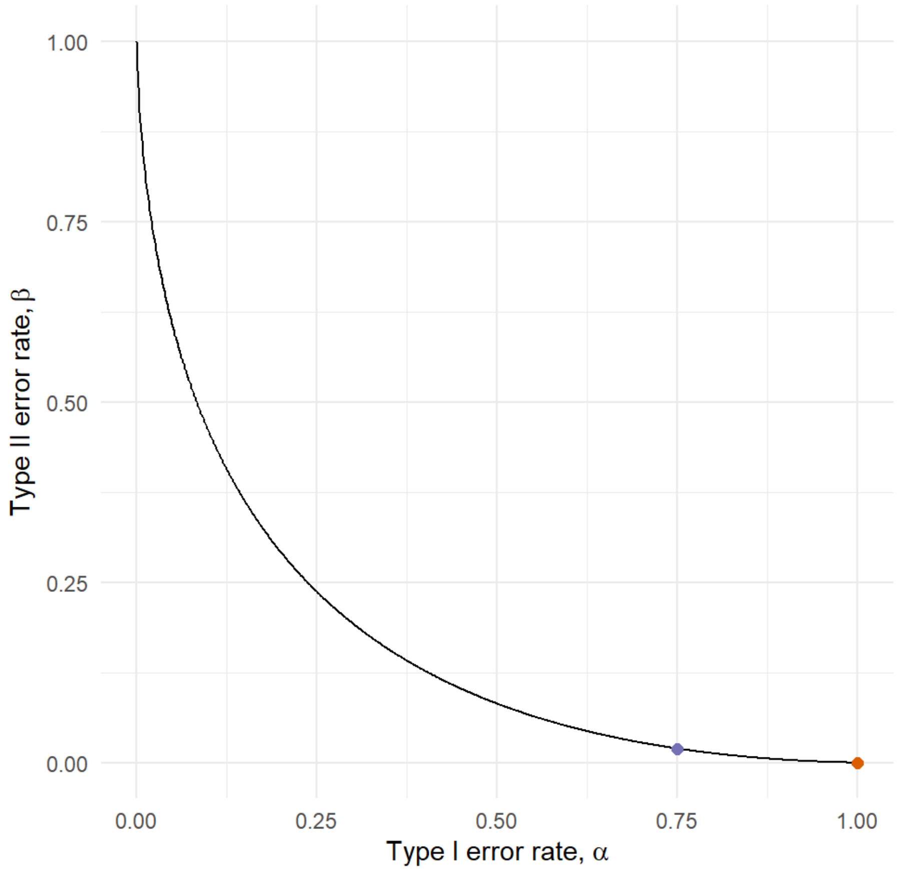


```
#ggsave("./figures/ocs.pdf", height=9, width=11, units="cm")
#ggsave("./figures/ocs.eps", height=9, width=11, units="cm", device = cairo_ps())
```
